# Supplementary material for: Impact of human papillomavirus age-related prevalence and vaccination levels on interpretation of cervical screening modalities: a modelling study
Source: BMJ Open. 2024 Feb 2;14(2):e078551. doi: 10.1136/bmjopen-2023-078551 (PMC10840029; doi:10.1136/bmjopen-2023-078551)
Supplement: Supplementary data [file bmjopen-2023-078551supp001.pdf]

Impact of Human Papillomavirus age-related prevalence and vaccination levels on interpretation of cervical screening modalities – a modelling study: Supplemental material

Author: David Robert Grimes[1,2], PhD

Addresses and affiliations:

1. School of Physical Sciences, Dublin City University, Glasnevin, Dublin, Ireland (davidrobert.grimes@dcu.ie)
2. Discipline of radiation therapy, Trinity College Dublin, Trinity Centre for Health Sciences, St. James's Hospital, Dublin 8, James's Street (davidrobert.grimes@tcd.ie)

40% Vaccination uptake simulation

Results as shown in figure 1.

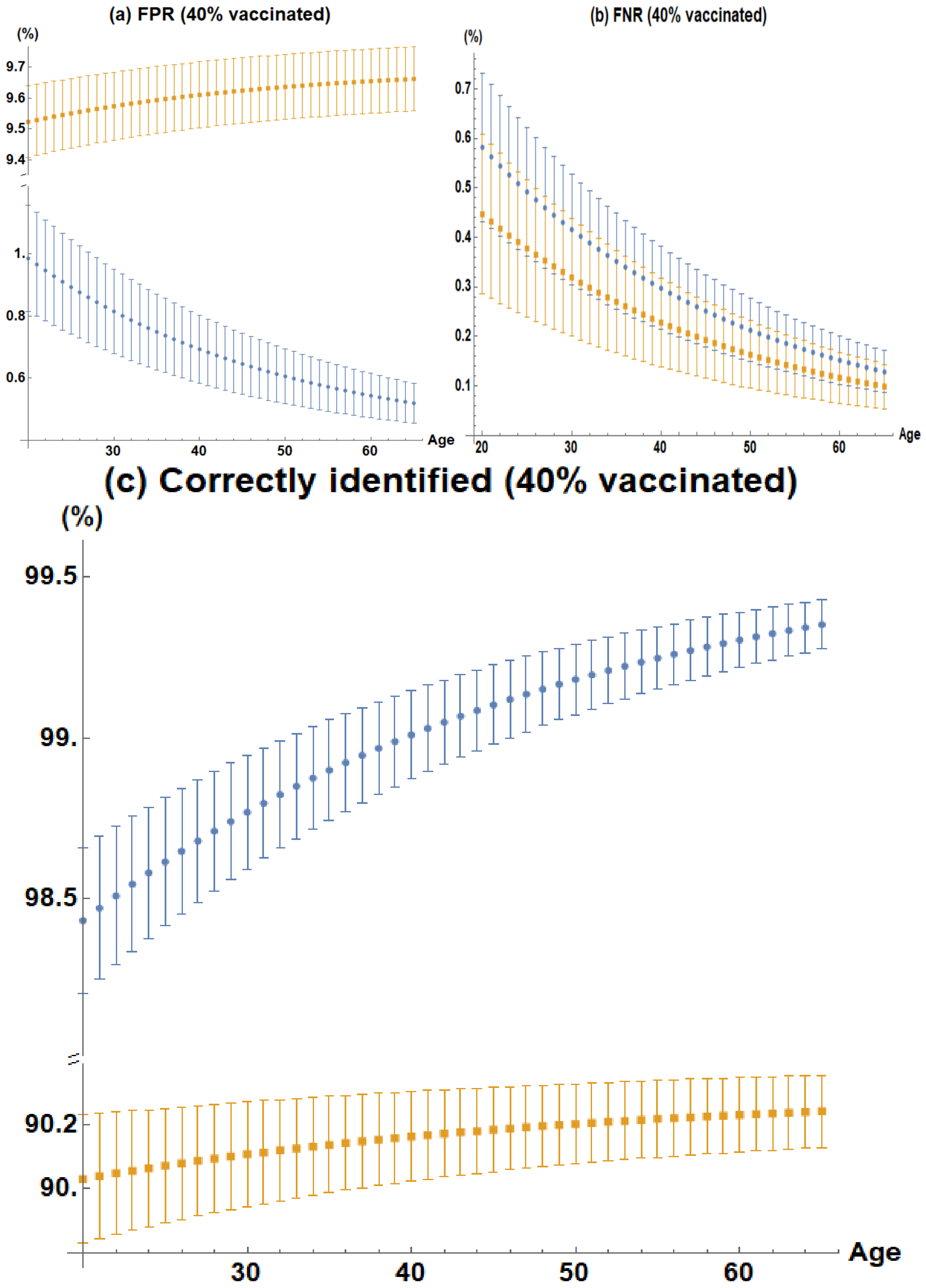

Figure 1 - 40% Vaccine uptake effect on (a) False positives (b) false negatives (c) correct identification

Age cohorts, sample size, and High risk HPV types

Study name	Number (n)	Age cohorts (N)	Hr- HPV types sampled
**Baay MF, Smits E, Tjalma WA, et al. Can cervical cancer screening be stopped at 50? The prevalence of HPV in elderly women.Int J Cancer 2004;108(2):258–61.**	1936	50-54, 55-59, 60-64, 65-69, 70+	16, 18, 31, 33, 35, 39, 45, 51, 52, 56, 58, 59, 66, 68.
**Vandenvelde C, Van Beers D. High-risk genital papillomaviruses and degree of dysplastic changes in the cervix: a prospective study by fast multiplex polymerase chain reaction in Belgium. J Med Virol 1993; 39(4):273**	323	20-30,31-40,41-50,51-60, 61-70	16,18,33
**Baay MF, Tjalma WA, Lambrechts HA, et al. Combined Pap and HPV testing in primary screening for cervical abnormalities:should HPV detection be delayed until age 35? Eur J Cancer 2005; 41(17):2704–8.**	2293	20-24, 25-29, 30-34, 35-39, 40-44, 45-50	16, 18, 31, 33, 35, 39, 45, 51, 52, 56, 58, 59, 66, 68
**Kjaer SK, van den Brule AJ, Bock JE, et al. Determinants for genital human papillomavirus (HPV) infection in 1000 randomly chosen young Danish women with normal Pap smear: are there different risk profiles for oncogenic and nononcogenic HPV types? Cancer Epidemiol Biomarkers Prev 1997;6(10):799–805**	956	20-23, 24-26, 27-29	16, 18, 31, 33, 35, 39, 45, 51, 52, 54, 56, 58, 59, 66, 68
**Boulanger JC, Sevestre H, Bauville E, et al. [Epidemiology of HPV infection]. Gynecol Obstet Fertil 2004;32(3):218–23**	3832	20-24, 25-29, 30-34, 35-39, 40-44,45-49, 50-54,55-59,60-64	16, 18, 31, 33, 35, 39, 45, 51, 52, 56, 58, 59 , 68
**Clavel C, Bory JP, Rihet S, et al. Comparative analysis of human papillomavirus detection by hybrid capture assay and routine cytologic screening to detect high-grade cervical lesions. Int J Cancer 1998; 75(4):525–8**	1028	14-20, 20-29, 30-39, 40-49, 50-59, 60-68	16, 18, 31, 33, 35, 45, 51, 52, 56
**Levert M, Clavel C, Graesslin O, et al. Human papillomavirus typing in routine cervical smears. Results from a series of 3778 patients]. Gynecol Obstet Fertil 2000;28(10):722–8**	3778	15-20, 21-30, 31-40, 41-50, 51-60	16, 18, 31, 33, 35, 39, 45, 51, 52, 56, 58, 59, 68
**Clavel C, Masure M, Bory JP, et al. Human papillomavirus testing in primary screening for the detection of high-grade cervical lesions: a study of 7932 women. Br J Cancer 2001;84(12):1616–23.**	7932	15-20, 21-30, 31-40, 41-50, 51-60	16, 18, 31, 33, 35, 39, 45, 51, 52, 56, 58, 59, 68
**Ronco G, Ghisetti V, Segnan N, et al. Prevalence of human papilloma- virus infection in women in Turin, Italy. Eur J Cancer 2005;41(2): 297–305.**	1013	25-29, 30-34, 35-39, 40-44, 45-49, 50-54, 55-59, 60-64	16, 18, 26, 31, 33, 34, 35, 39, 40, 42, 43, 44, 45, 51–59, 61, 66, 68, 70, 71, 72, 73, 81, 82, 83, 84, CP6108
**Jacobs MV, Walboomers JM, Snijders PJ, et al. Distribution of 37 mucosotropic HPV types in women with cytologically normal cervical**	3305	15-24, 25-29, 30-34, 35-39, 40-44, 45-49, 50-54, 55-	16, 18, 31, 33, 35, 39, 45, 51, 52, 56, 58, 59, 66, 68

**smears: the age-related patterns for high-risk and low-risk types. Int J Cancer 2000;87(2):221–7.**		59, 60-69	
**Cuzick J, Szarewski A, Terry G, et al. Human papillomavirus testing in primary cervical screening. Lancet 1995;345(8964):1533–6.**	2007	15-25, 25-29, 30-34, 35-39, 40-44, 45-49, 50-54, 55-69	16, 18, 31, 33
**Peto J, Gilham C, Deacon J, et al. Cervical HPV infection and neoplasia in a large population-based prospective study: the Manchester cohort. Br J Cancer 2004;91(5):942–53**	6128	15-19, 20-24, 25-29, 30-34, 35-39, 40-44, 45-49, 50-54, 55-69	16, 18, 26, 31, 33, 35, 39, 40, 45, 51, 52, 53, 54, 55, 56, 57, 58, 59, 66, 68, 73, 82, 83 84

Mathematical justifications for model assumptions

We can show that CIN2+ prevalence will be directly proportional to HPV prevalence, even accounting for strain types and their respective likelihood of causing lesions. Consider n strains of HPV, each constituting a proportion p of the total HPV burden. The HPV burden may be written as

$$H = p_1 + p_2 \ldots + p_n = \sum_1^n p_n.$$

We may write p as a vector, and defining u as a vector of length n with every entry equal to unity, we note through the properties of the dot product operator that it follows that

$$H = p \cdot u = \sqrt{n}|p| \cos \emptyset \rightarrow |p| = \frac{H}{\sqrt{n} \cos \emptyset}$$

where $\emptyset$ is the angle between the vectors. We can further state that each strain of HPV has a probability of becoming a CIN2+ lesion of $c_i$respectively per strain, ranging from 0 to 1. Thus, the total lesion burden is given by

$$C = c_1 p_1 + c_2 p_2 \ldots + c_n p_n = \sum_1^n c_n p_n.$$

The values for p and c can be vectors and from the properties of the dot product, we may write

$$C = p \cdot c = \ |p||c| \cos \theta = \frac{H\ |c| \cos \theta}{\sqrt{n} \cos \emptyset}.$$

where θ is the angle between the prevalence and risk vectors. As can be seen here, it follows that no matter the configuration of these vectors, $C \propto H$ and so the assumption is justified, regardless of the individual strains involved. The cited figure for HPV prevalence after vaccination in this work is empirical, derived from previous modelling studies, functioning simply as a multiplier to gauge the performance of HPV-reflex screening under reduced incidence of infection, independent of strain. We can however use a similar argument to the previous response to show here why the approximately linear correspondence holds. Let v be the vector efficacy of vaccination for each strain, with each entry given by

$$v_i = (1 - e_i)$$

where e_i is the fractional respective efficacy of the vaccine against each strain, bounded between 0 and unity. We denote an element-by-element Hadamard product, corresponding to the reduced prevalence of each HPV subtype after vaccination as $p_v = p \odot v$, and like before, the modified HPV prevalence after vaccination is given by $H_v = p_v \cdot u$. But as the Hadamard product is commutative, we can instead write this as $H_v = p \cdot (u \odot v)$. Letting $u \odot v = u_v$ for brevity, then

$$H_v = p \cdot u_v = \sqrt{u_v}|p| \cos \emptyset_{uv} \rightarrow |p| = \frac{H_v}{\sqrt{u_v} \cos \emptyset_{uv}}.$$

This can be set equal to the value for $|p|$ previously derived and rearranged to establish the identity

$$H_v = H\left(\frac{\sqrt{u_v} \cos \emptyset_{uv}}{\sqrt{n} \cos \emptyset}\right).$$

The bracketed term is effectively a constant, corresponding to the reduction in net HPV infection. This is akin to the $(1 - V)$ term in the work itself, demonstrating the empirical use of a linear reduction factor derived from previous studies is justified to ascertain the performance of screening modalities under different levels of coverage.

Model performance under inflated $\bar{h}$

In this work, the population prevalence of HPV is experimentally derived, yielding $\bar{h} = 9.4 \pm 2.3$ %, in good agreement with literature estimate of 8.4%, and with population wide European estimates of 9.8% (95% Confidence Interval: 9.2%-10.0%) and more recent results from the ATHENA study. Estimates in literature however can be someway complicated by how sampling conducted. In this model, $\bar{h}$ is data derived, but we can also force a higher value into the simulation to examine consistency ever with much higher prevalence to ascertain how screening modalities would perform with $\bar{h} = 15\%$ to investigate this. The table below shows correct, false positive, and false negative results for different age groups under both the data derived and forced HPV prevalence scenarios. As can be seen from the table, test performance results are broadly similar under both data derived and forced scenarios.

Screening accuracy per 1000 women

	$\bar{h} = 9.4 \mp 2.3\%$ (Data derived)			$\bar{h} = 15\%$ (Forced)		
**Age (modality)**	Correct	False positives	False Negatives	Correct	False positives	False Negatives
**25 (LBC)**	898 ∓ 3	94 ∓ 1	8 ∓ 3	900 ∓ 2	95 ∓ 1	5 ∓ 1
**25 (HPV-reflex)**	975 ∓ 4	15 ∓ 3	11 ∓ 3	978 ∓ 3	16 ∓ 3	7 ∓ 1
**35 (LBC)**	900 ∓ 2	95 ∓ 1	6 ∓ 2	901 ∓ 1	96 ∓ 1	4 ∓ 1
**35 (HPV-reflex)**	981 ∓ 3	12 ∓ 2	8 ∓ 2	983 ∓ 2	12 ∓ 2	5 ∓ 1
**45 (LBC)**	901 ∓ 2	95 ∓ 1	4 ∓ 2	901 ∓ 1	96 ∓ 1	3 ∓ 1
**45 (HPV-reflex)**	985 ∓ 2	9 ∓ 1	5 ∓ 2	989 ∓ 2	10 ∓ 2	3 ∓ 1
**55 (LBC)**	901 ∓ 2	96 ∓ 1	3 ∓ 1	902 ∓ 1	96 ∓ 1	2 ∓ 1
**55 (HPV-reflex)**	988 ∓ 3	8 ∓ 2	4 ∓ 1	989 ∓ 2	8 ∓ 2	2 ∓ 1
