# Supplementary material for: Impact of human papillomavirus age-related prevalence and vaccination levels on interpretation of cervical screening modalities: a modelling study
Source: BMJ Open. 2024 Feb 2;14(2):e078551. doi: 10.1136/bmjopen-2023-078551 (PMC10840029; doi:10.1136/bmjopen-2023-078551)
Supplement: Author's manuscript [file bmjopen-2023-078551.draft_revisions.pdf]

BMJ Open is committed to open peer review. As part of this commitment we make the peer review history of every article we publish publicly available.

When an article is published we post the peer reviewers' comments and the authors' responses online. We also post the versions of the paper that were used during peer review. These are the versions that the peer review comments apply to.

The versions of the paper that follow are the versions that were submitted during the peer review process. They are not the versions of record or the final published versions. They should not be cited or distributed as the published version of this manuscript.

BMJ Open is an open access journal and the full, final, typeset and author-corrected version of record of the manuscript is available on our site with no access controls, subscription charges or pay-per-view fees (http://bmjopen.bmj.com).

If you have any questions on BMJ Open's open peer review process please email info.bmjopen@bmj.com

BMJ Open

Impact of Human Papillomavirus age-related prevalence and vaccination levels on interpretation of cervical screening modalities – a modelling study

Journal:	*BMJ Open*
Manuscript ID	bmjopen-2023-078551
Article Type:	Original research
Date Submitted by the Author:	04-Aug-2023
Complete List of Authors:	Grimes, David; University of Oxford, & Queens University Belfast
Keywords:	Mass Screening, PUBLIC HEALTH, Public health < INFECTIOUS DISEASES, Sexually Transmitted Disease

SCHOLARONE™
Manuscripts

I, the Submitting Author has the right to grant and does grant on behalf of all authors of the Work (as defined in the below author licence), an exclusive licence and/or a non-exclusive licence for contributions from authors who are: i) UK Crown employees; ii) where BMJ has agreed a CC-BY licence shall apply, and/or iii) in accordance with the terms applicable for US Federal Government officers or employees acting as part of their official duties; on a worldwide, perpetual, irrevocable, royalty-free basis to BMJ Publishing Group Ltd ("BMJ") its licensees and where the relevant Journal is co-owned by BMJ to the co-owners of the Journal, to publish the Work in this journal and any other BMJ products and to exploit all rights, as set out in our licence.

The Submitting Author accepts and understands that any supply made under these terms is made by BMJ to the Submitting Author unless you are acting as an employee on behalf of your employer or a postgraduate student of an affiliated institution which is paying any applicable article publishing charge ("APC") for Open Access articles. Where the Submitting Author wishes to make the Work available on an Open Access basis (and intends to pay the relevant APC), the terms of reuse of such Open Access shall be governed by a Creative Commons licence – details of these licences and which Creative Commons licence will apply to this Work are set out in our licence referred to above.

Other than as permitted in any relevant BMJ Author's Self Archiving Policies, I confirm this Work has not been accepted for publication elsewhere, is not being considered for publication elsewhere and does not duplicate material already published. I confirm all authors consent to publication of this Work and authorise the granting of this licence.

For peer review only

Impact of Human Papillomavirus age-related prevalence and vaccination levels on interpretation of cervical screening modalities – a modelling study

Author: David Robert Grimes[1,2], PhD

Addresses and affiliations:

1. Discipline of radiation therapy, Trinity College Dublin, Trinity Centre for Health Sciences, St. James's Hospital, Dublin 8, James's Street (davidrobert.grimes@tcd.ie)
2. School of Physical Sciences, Dublin City University, Glasnevin, Dublin, Ireland (davidrobert.grimes@dcu.ie)

Word count: 2500

Figure count: 3

Table count: 2

For peer review only

Impact of Human Papillomavirus age-related prevalence and vaccination levels on interpretation of cervical screening modalities – a modelling study

David Robert Grimes, PhD –davidrobert.grimes@tcd.ie

For peer review only

Abstract

Objective: Cervical screening is a life-saving intervention, and Human Papillomavirus (HPV) based screening modalities hold unique promise in improving screening accuracy. HPV prevalence varies markedly by age, as does resultant cervical intra-epithelial neoplasia (CIN), with higher rates recorded in younger women. With the advent of effective vaccination for HPV drastically reducing prevalence of both HPV and CIN, it is critical to model how the accuracy of different screening approaches varies with age-cohort and vaccination status. This work establishes a model for age-specific prevalence of HPV factoring in vaccine coverage, and predicts how the accuracy of common screening modalities is affected by age profile and vaccine uptake.

Design: Modelling study of HPV infection rates by age, ascertained from European cohorts prior to the introduction of vaccination. Reductions in HPV due to vaccination were estimated from the bounds predicted from multiple modelling studies, yielding a model for age-varying HPV and CIN grades 2 and above (CIN2+) prevalence.

Setting: Performance of both conventional Liquid-base cytology (LBC) screening and HPV screening with LBC reflex were estimated under different simulated age cohorts and vaccination levels.

Participants: Simulated populations of varying age and vaccination status

Results: HPV-reflex modalities consistently result in much lower incidence of false positives than LBC testing, with an accuracy that improves even as HPV and CIN2+ rates decline.

Conclusions: HPV-reflex tests outperform LBC tests across all age profiles, resulting in greater test accuracy. This improvement is especially pronounced as HPV infection rates fall and suggests HPV-reflex modalities are robust to future changes in the epidemiology of HPV.

Strengths and weaknesses

- Extends modelling approaches to screening outcomes by incorporating the projected HPV prevalence and vaccine uptake levels of age-varying cohort.
- Provides a novel methodology for ascertaining how screening modalities will perform under likely future scenarios.
- HPV prevalence estimates are limited by assumptions efficacy and uptake of vaccination, and this can be varied with the model presented.

Introduction

Cervical cancer screening is a powerful life-saving intervention, associated with significant reductions in the mortality and cancer burden[1-3]. For squamous cell carcinoma in women older than 25 years,

national screening paradigms have resulted in an estimated 80% reduction in mortality. Human papillomavirus (HPV) is responsible for most of these cancers, and the advent of vaccination against its oncogenic strains has already had transformative impact on both rates of HPV and cervical intra-epithelial neoplasia (CIN)[2] .

Alongside conventional Liquid based cytology (LBC) modalities for screening for CIN grades 2 and higher (CIN2+), the evolution of screening modalities to include HPV-reflex method which triage cases by HPV infection status have shown great promise[4-6]. This promise however requires careful interpretation - HPV testing is often deemed to have higher sensitivity than conventional LBC approaches, but there are nuances which demand careful consideration. The sensitivity of HPV tests is not a direct proxy to LBC sensitivity, as they measure different things. While LBC tests directly measure the presence of CIN2+, HPV tests themselves only detect the presence of high-risk strains of HPV. In younger women especially, there is high transient incidence of HPV, and using HPV infection alone as a screening modality would result in an unacceptably high false detection rate of CIN2+.

Instead, HPV triage approaches are typically implemented, where patients testing positive for high-risk HPV receive a reflex LBC, while those testing negative do not. A recent analysis[3] demonstrated that this reflex approach has in fact a slightly lower effective sensitivity for CIN2+ (0.68, 95% Confidence interval: 0.60-0.75) than LBC testing alone (0.76, 95% Confidence interval: 0.67-0.83) in a typical population, though this seeming disadvantage is typically mitigated by expedited retesting of HPV positive results before the next screening cycle. The chief advantage of HPV-reflex tests (or LBC tests with HPV reflex) is that they hugely decrease the false positive rate, with a reflex test yielding 9.6 false positives per 1000 women (95% Confidence interval: 9.4-9.8) compared to LBC testing with 95.1 false positives per 1000 women (95% Confidence interval: 93.7- 97.0), decreasing false positive rates by approximately ten-fold and reducing overtreatment. Simplified clinical patient flow for both LBC and HPV reflex modalities are illustrated in figure 1.

Yet to date, this work has been largely considered average HPV infection rates and CIN2+ prevalence averaged across entire populations. HPV infection rates however differ by both age cohort[7] and vaccination status[8]. Younger women tend to have higher rates of both HPV infection, and CIN2+. This in turn has implications for current and future screening interpretations and planning with differing cohorts. CIN2+ prevalence is directly proportional to HPV infection[9] and will change with as infection rates change either due to aging cohorts or increased vaccine uptake. A simple dynamic model to account for this would allow accurate determination of screening performance under realistic scenarios and facilitate the planning of future screening modalities. This work accordingly establishes a model to account HPV infection rates, and CIN2+ prevalence with age cohorts and vaccine uptake, ascertaining test accuracy under all possible future scenarios.

Figure 1 - Clinical patient flow for (a) LBC screening and (b) HPV reflex screening. HPV positive, LBC negative results are frequently selected for an expedited retest outside of the normal screening interval

Methods

Modelling varying prevalence of HPV and CIN2+

HPV infection rates vary markedly by country and cohort, and even detection methodology. The evidence to date strongly suggests that HPV infection peaks before 30, though slightly different trends emerge in various data sets. For the purposes of this investigation, analysis was restricted to sets prior to widespread coverage of the HPV vaccine. On current estimates from USA and European data, we expect a global average of approximately 8.4% of the total screened population to have active HPV-infection at the time of screening[3].

For peer review only

For this work, it was crucial to further stratify this figure by age cohort. To derive an age-specific HPV prevalence rate, we considered the review by Smith et al[7] (2008) of the literature on HPV infection rates throughout the world. This review included various data sources, including these stemming from sexual infection clinics or specific high-risk populations. As these results are likely to produce high (or in some cases low) outliers which can skew analysis, we confined ourselves only to the subset of studies solely from general European screening programmes. Furthermore, this analysis only considered studies with some degree of age stratification. This resulted in 12 applicable studies[10-21]. These studies were undertaken in various cities throughout Belgium, Denmark, France, Italy, The Netherlands, and the United Kingdom between 1992 and 2005, with samples ranging from 323 women to 7932 women (mean: 2878 women). Data from these studies was then taken and weighted according to the number of study participants and smoothed, so that an age-varying phenomenological model describing HPV prevalence at age in years *a* could be ascertained with 95% confidence intervals, denoted $h_u(a)$. Details of these studies, participants, settings, and HPV subtypes tested for are given in the supplementary material.

Crucially, these estimates stem from before the advent of HPV vaccination, and accordingly map to a scenario of 0% vaccine coverage. This is a useful baseline upon which we can model the impact of vaccination on test performance. The impact of HPV vaccination on prevalence rates was derived from 29 previously published modelling studies[8], yielding an estimate for *V*, the reduction factor for HPV prevalence from given level of vaccination, as previously published[3,8]. The time-varying HPV prevalence for a given level of vaccination is thus $h(a) = (1 - V)h_u(a)$. For brevity, results in this work consider the impact of vaccine rates of 0% (completely unvaccinated cohorts) and 80% (highly vaccinated cohorts) with mid-level vaccination rates of 40% given in the supplementary material. The mean value for $h(a)$ across the entire span of screening ages is given by

$$\bar{\mathrm{h}} = \frac{1}{\mathrm{a_e} - \mathrm{a_s}} \int_{a_s}^{\mathrm{a_e}} \mathrm{h}(a)\mathrm{da}\,.$$

Taking the upper bound of screening ages to be $\mathrm{a_e} = 65$ years and the lower bound to be $\mathrm{a_s} = 20$ years, the mean HPV prevalence in the cohort can be ascertained from the form of the phenomenological function. Under the reasonable assumption that CIN2/3 prevalence is proportional to the rates of HPV infection, it follows that a simple function of scaling should be predictive of prevalence rates for CIN2+. If the known population average of CIN2+ is $\bar{\mathrm{p}}$, then age / vaccine varying CIN2+ prevalence is accordingly given by

$$p(a) = \left(\frac{\bar{p}}{\bar{h}}\right)h(a).$$

Interpretation of screening results with varying HPV prevalence and vaccine uptake.

With age and vaccination specific CIN2/3 prevalence known, one can explicitly calculate false positive rates, false negative rates, and over-all correct identifications using previously published methods. For Liquid based cytology (LBC), the false positive (fp_l), false negative (fn_l), and over-all correct identification rates (t_l) are related to CIN2/3 prevalence and the sensitivity (s_{nl}) and specificity (s_{pl}) of LBC testing, given respectively by the relationships

$$fp_l(a) = (1 - p(a))(1 - s_{pl})$$

$$fn_l(a) = p(a)(1 - s_{nl})$$

For peer review only

$$t_l(a) = 1 - (f_{pl}(a) + f_{nl}(a)).$$

For HPV reflex testing, false positive rates (fp_r), false negative rates (fn_r),, and over-all correct identification rate (t_r) depend additionally on HPV prevalence, the sensitivity of HPV testing (s_{nh}), the specificity of HPV testing (s_{ph}) and the fraction of CIN2+ cases associated with detectable HPV, v. These equations are derived from those previously published[3], and are respectively

$$fp_r(a) = (1 - s_{pl})(s_{nh}(h(a) - p(a)v) + (1 - s_{ph})(1 - h(a) - p(a)(1 - v)))$$

$$fn_r(a) = vp(a)(1 - s_{nl}s_{nh}) + p(a)(1 - v)(1 - s_{nl}(1 - s_{ph}))$$

$$t_r(a) = 1 - (f_{pl}(a) + f_{nl}(a)).$$

Literature values used for all modelling simulations are given in table 1.

Table 1: Simulation values and source

Parameter	Values (95% CI where available)
Average prevalence of CIN2/3 in full population ($\bar{p}$)	0.026[6]
Sensitivity of LBC test to CIN2+ (s_{nl})	0.76 ± 0.04 [6]
Specificity of LBC test to cin2+ (s_{pl})	0.903 ± 0.001 [6]
Sensitivity of HPV test to High-Risk HPV (s_{nh})	$0.947\ (-)$ [3]
Specificity of HPV test to High-Risk HPV (s_{ph})	0.96 ± 0.001 [6]
CIN2/3 attributable to testable hr-HPV (v)	$0.95(-)$ [3]
Reduction in HPV at 40% vaccine coverage (V_{40})	0.53 ± 0.06 [3,8]
Reduction in HPV at 80% vaccine coverage (V_{80})	0.93 ± 0.026 [3,8]

Patient and Public Involvement

None. It was not appropriate or possible to involve patients or the public in the design, or conduct, or reporting, or dissemination plans of this research.

Results

Modelling varying prevalence of HPV and CIN2+

Age-related HPV prevalence from 12 included studies were found to approximately obey an exponential decay type-model from ages 20-65 of the form $A\exp(-\beta t)$ as illustrated in figure 1 ($A = 35.81 \pm 4.91,\ \beta = 0.0336 \pm 0.0045\ yr^{-1}$, 95% confidence interval). This envelope with

weighed study data points are shown in figure 1. The estimate for mean HPV infection rate in unvaccinated populations across all age ranges was $\bar{h} = 9.4 \pm 2.3\,\%$, in good agreement with literature estimate of 8.4%. CIN2+ rates by age cohort from inferred from this as per the model outline.

Figure 2 - Weighted fits from 12 European population studies with exponential fit and confidence intervals.

Interpretation of screening results with varying HPV prevalence and vaccine uptake

Figure 2 depicts how the accuracy of LBC and HPV-reflex modalities (in terms of true results, false positives, and false negatives) change with age in both unvaccinated and 80% vaccinated populations. HPV reflex modalities consistently outperform LBC modalities for all age cohorts and vaccine uptake levels. Table 2 depicts a selection of results for varying age profiles and vaccine uptake, with additional Simulation results for 40% vaccine coverage are given in the supplementary material.

Figure 3: Performance of HPV-reflex and LBC screening modalities with age. Percentage correct results shown for (a) unvaccinated populations and (b) 80% vaccine uptake populations. False positive rates of both modalities with age shown for (c) unvaccinated populations and (d) 80% vaccine uptake populations. False negative rates depicted for (e) unvaccinated populations and (f) 80% vaccinated populations. Note the broken Y-axis in (a)-(d) inclusive

Table 2: Modelled test performance at specific ages and vaccination levels

	LBC			HPV reflex		
**Age profile**	***False Negative %***	***False Positive %***	***Correct Result %***	***False Negative %***	***False Positive %***	***Correct Result %***
Age 25						
**0% Vaccinated**	0.8 ± 0.28	9.38 ± 0.14	89.1 ± 0.31	1.05 ± 0.26	1.46 ± 0.27	97.50 ± 0.40
**40% Vaccinated**	0.38 ± 0.14	9.55 ± 0.11	90.07 ± 0.18	0.49 ± 0.13	1.61 ± 0.26	97.89 ± 0.29
**80% Vaccinated**	0.06 ± 0.03	9.68 ± 0.10	90.27 ± 0.11	0.07 ± 0.03	1.73 ± 0.26	98.20 ± 0.26
Age 35						
**0% Vaccinated**	0.57 ± 0.21	9.47 ± 0.12	89.95 ± 0.24	0.75 ± 0.20	1.16 ± 0.23	98.10 ± 0.30
**40% Vaccinated**	0.27 ± 0.10	9.59 ± 0.11	90.14 ± 0.15	0.35 ± 0.10	1.26 ± 0.22	98.38 ± 0.24
**80% Vaccinated**	0.04 ± 0.02	9.68 ± 0.10	90.28 ± 0.10	0.05 ± 0.02	1.35 ± 0.22	98.60 ± 0.22
Age 45						
**0% Vaccinated**	0.41 ± 0.16	9.54 ± 0.12	90.05 ± 0.20	0.53 ± 0.15	0.94 ± 0.19	98.53 ± 0.24
**40% Vaccinated**	0.19 ± 0.08	9.62 ± 0.11	90.18 ± 0.11	0.25 ± 0.07	1.01 ± 0.18	98.73 ± 0.20
**80% Vaccinated**	0.03 ± 0.02	9.69 ± 0.10	90.28 ± 0.10	0.04 ± 0.01	1.07 ± 0.18	98.89 ± 0.18
Age 55						
**0% Vaccinated**	0.29 ± 0.12	9.58 ± 0.11	90.12 ± 0.16	0.38 ± 0.11	0.78 ± 0.16	98.84 ± 0.19
**40% Vaccinated**	0.14 ± 0.06	9.65 ± 0.10	90.22 ± 0.12	0.18 ± 0.06	0.84 ± 0.15	98.99 ± 0.16
**80% Vaccinated**	0.02 ± 0.01	9.69 ± 0.10	90.29 ± 0.10	0.03 ± 0.01	0.88 ± 0.15	99.10 ± 0.10

For peer review only

Discussion

In all simulated cases with realistic parameters, HPV-reflex testing correctly identifies a higher proportion of cases $(97.1 \pm 0.4\%$ to $99.6 \pm 0.2\%)$ relative to LBC testing for the same scenarios ($89.7 \pm 0.4\%$ to $90.3 \pm 0.1\%$). This is seen clearly in Figure 3(a) and (b), and detailed in table 2. The superior performance of HPV-reflex manifests chiefly due to markedly lower false positive rates. In all scenarios, LBC false positive rates exceed HPV-reflex values for all scenarios by a factor of 5.6 to 23.8. This analysis focused on a comparison of HPV-reflex to LBC testing. It did not explicitly consider co-testing, where both LBC and HPV screening are performed and a positive on either leads to colposcopy, as this modality leads to unacceptably high false positives results, although it is possible in principle to extend the analysis here to consider how this scenario would vary by age cohort and vaccination status.

This difference is particularly pronounced in vaccinated cohorts, as false positives decline markedly with lower HPV prevalence in HPV-reflex testing. By contrast, false positive rates slightly increase in vaccinated cohorts with LBC testing, as seen in figure 3(c)-(d). This Is expected, as the limit of LBC false positive rate as CIN2+ prevalence approaches zero is simply $1 - s_{pl}$, yielding a theoretical maximum possible false positive rate for LBC of approximately 9.7%. By contrast, false positives decline with reflex with HPV, resulting in a theoretical minimum false positive rate of $(1 - s_{pl})(1 - s_{ph})$, or a minimum value of approximately 0.39%.

For both LBC and reflex testing, false negatives decline with decreasing HPV prevalence. LBC testing does have a slightly lower false negative rate than HPV-reflex testing, but this is marginal and overlaps with the performance envelope for HPV-reflex testing. In principle, LBC testing is more sensitive to instances of CIN2+ than reflex screening, but it results in far more false positives and needless referrals to colposcopy. As HPV detection sensitivity is so high, expediated retesting can be employed when a patient tests positive for a high-risk strain of HPV, and negative on LBC. This is likely to more than compensate for the marginally higher false negative rate of reflex testing, while preserving the crucial benefit and over-all accuracy. Expedited retesting is already implemented for HPV positive results in several screening programmes, including the Irish National screening programme CervicalCheck[22] . where HPV positive but cytology negative patients are recalled for repeat screening after one year rather than three or five. Such approaches maintain the benefits of HPV-reflex without the potential drawback of lower net-sensitivity.

One limitation of the current study was the assumption that CIN2+ prevalence corresponds linearly to HPV prevalence, yielding an approximately constant proportion with age cohort. This is a reasonable assumption, but there are several important caveats that need to be considered. Firstly, the proportion estimated in this work pivots on how well the populations in the HPV and CIN2+ datasets correspond to one another. While these data sets were chosen to avoid the potentially skewing influence of elective sexually transmitted disease clinic settings, there may be other confounding reasons why they are not representative, in which case estimates of $p(a)$ might be biased.

In addition, the proportionality itself might vary with time for biological reasons. While younger women for example might have a higher rate of HPV, it is conceivable that they have better mechanisms of clearance, and so proportionally might have a lower fraction of CIN2+ compared to older cohorts. In the absence of clear data, this is of course speculative but the existence of any such age-specific variation in clearance rate would potentially impact the estimates in this work.

It is important to note that this analysis did not consider the impacts of follow-up between screening rounds, nor the effects intervals between screening rounds, yielding instead an age-specific point

For peer review only

estimate of false positive and negative rates in isolation. The cumulative false positive and negative rate over multiple screening rounds will vary depending on the intervals between screening, the rate of HPV clearance, and the modalities employed. This is an involved question beyond the scope of this work, and the impacts of repeated screening on false negative and positive rates require dedicated future analysis to answer fully.

In consequence, HPV-reflex testing is calculated to be more robust to changing age and vaccine status than LBC testing and will increase further in accuracy as HPV infection decreases due to vaccination. This is an important consideration for the planning and interpretation of future screening, which need to be robust for both age variation of HPV infection, and the eventuality where HPV infection is virtually eradicated due to increased uptake of the HPV vaccine. The results of this analysis strongly suggest that reflex screening approaches are robust to changing epidemiology of HPV infection, and will continue to perform strongly even as HPV infections fall with increasing vaccine uptake.

Competing interests statement

Dr Grimes reported that he has written articles on both screening and HPV vaccination for popular audiences, receiving no industry funding for these articles. He also reports that he has consulted for public health bodies and charities in Europe on screening. He also reported receiving paid royalties for a popular science book and receiving fees and honoraria for contributed articles, fact-checking, public talks, and media appearances.

Ethical approval statement

This work involved no patient or personal data and only used publicly available and previously published prevalence data. Accordingly, no ethical approval was required for this work.

Funding statement

This work was funded by grant number 214461/Z/18/Z from the Wellcome Trust. The funder had no role in the design and conduct of the study; collection, management, analysis, and interpretation of the data; preparation, review, or approval of the manuscript; and decision to submit the manuscript for publication.

Data sharing statement

Source code for the simulations herein are hosted online at
https://github.com/drg85/CervScreenAgeVax

Authors' contributions

DRG conceived the model, analysed the published data, performed the analysis, reported the results and wrote the manuscript.

References

1. Peto, J., Gilham, C., Fletcher, O., & Matthews, F. E. (2004). The cervical cancer epidemic that screening has prevented in the UK. The Lancet, 364(9430), 249-256.

For peer review only

2. Hall, M. T., Simms, K. T., Lew, J. B., Smith, M. A., Brotherton, J. M., Saville, M., ... & Canfell, K. (2019). The projected timeframe until cervical cancer elimination in Australia: a modelling study. The Lancet Public Health, 4(1), e19-e27.
3. Grimes, D. R., Corry, E. M., Malagón, T., O'Riain, C., Franco, E. L., & Brennan, D. J. (2021). Modeling cervical cancer screening strategies with varying levels of human papillomavirus vaccination. JAMA network open, 4(6), e2115321-e2115321.
4. Franco EL, Mahmud SM, Tota J, Ferenczy A, Coutlée F. The expected impact of HPV vaccination on the accuracy of cervical cancer screening: the need for a paradigm change. Arch Med Res. 2009;40(6):478-485
5. Ronco G, Dillner J, Elfström KM, et al; International HPV screening working group. Efficacy of HPV-based screening for prevention of invasive cervical cancer: follow-up of four European randomised controlled trials. Lancet. 2014;383(9916):524-532. doi:10.1016/S0140-6736(13)62218-7
6. Koliopoulos G, Nyaga VN, Santesso N, et al. Cytology versus HPV testing for cervical cancer screening in the general population. Cochrane Database Syst Rev. 2017;8:CD008587. doi:10.1002/14651858.CD008587.pub2
7. Smith, J. S., Melendy, A., Rana, R. K., & Pimenta, J. M. (2008). Age-specific prevalence of infection with human papillomavirus in females: a global review. Journal of Adolescent Health, 43(4), S5-e1.
8. Brisson, M., Bénard, É., Drolet, M., Bogaards, J. A., Baussano, I., Vänskä, S., ... & Walsh, C. (2016). Population-level impact, herd immunity, and elimination after human papillomavirus vaccination: a systematic review and meta-analysis of predictions from transmission-dynamic models. The Lancet Public Health, 1(1), e8-e17.
9. Ting J, Kruzikas DT, Smith JS. A global review of age-specific and overall prevalence of cervical lesions. Int J Gynecol Cancer. 2010 Oct;20(7):1244-9. doi: 10.1111/igc.0b013e3181f16c5f. PMID: 21495248.
10. Baay MF, Smits E, Tjalma WA, et al. Can cervical cancer screening be stopped at 50? The prevalence of HPV in elderly women.Int J Cancer 2004;108(2):258–61.
11. Vandenvelde C, Van Beers D. High-risk genital papillomaviruses and degree of dysplastic changes in the cervix: a prospective study by fast multiplex polymerase chain reaction in Belgium. J Med Virol 1993; 39(4):273
12. Baay MF, Tjalma WA, Lambrechts HA, et al. Combined Pap and HPV testing in primary screening for cervical abnormalities:should HPV detection be delayed until age 35? Eur J Cancer 2005; 41(17):2704–8.
13. Kjaer SK, van den Brule AJ, Bock JE, et al. Determinants for genital human papillomavirus (HPV) infection in 1000 randomly chosen young Danish women with normal Pap smear: are there different risk profiles for oncogenic and nononcogenic HPV types? Cancer Epidemiol Biomarkers Prev 1997;6(10):799–805
14. Boulanger JC, Sevestre H, Bauville E, et al. [Epidemiology of HPV infection]. Gynecol Obstet Fertil 2004;32(3):218–23
15. Clavel C, Bory JP, Rihet S, et al. Comparative analysis of human papillomavirus detection by hybrid capture assay and routine cytologic screening to detect high-grade cervical lesions. Int J Cancer 1998; 75(4):525–8
16. Levert M, Clavel C, Graesslin O, et al. Human papillomavirus typing in routine cervical smears. Results from a series of 3778 patients]. Gynecol Obstet Fertil 2000;28(10):722–8
17. Clavel C, Masure M, Bory JP, et al. Human papillomavirus testing in primary screening for the detection of high-grade cervical lesions: a study of 7932 women. Br J Cancer 2001;84(12):1616–23.

For peer review only

18. Ronco G, Ghisetti V, Segnan N, et al. Prevalence of human papilloma- virus infection in women in Turin, Italy. Eur J Cancer 2005;41(2): 297–305.
19. Jacobs MV, Walboomers JM, Snijders PJ, et al. Distribution of 37 mucosotropic HPV types in women with cytologically normal cervical smears: the age-related patterns for high-risk and low-risk types. Int J Cancer 2000;87(2):221–7.
20. Cuzick J, Szarewski A, Terry G, et al. Human papillomavirus testing in primary cervical screening. Lancet 1995;345(8964):1533–6.
21. Peto J, Gilham C, Deacon J, et al. Cervical HPV infection and neoplasia in a large population-based prospective study: the Manchester cohort. Br J Cancer 2004;91(5):942–53
22. CervicalCheck National Screening Programme, Health Service Executive Ireland. Cervical Screening results and recommendations table CS/PUB/LAB-2 Rev. 12 (2021) – Available online from https://www.cervicalcheck.ie/_fileupload/Health-professionals/CervicalScreeningResultsandRecommendations.pdf

For peer review only

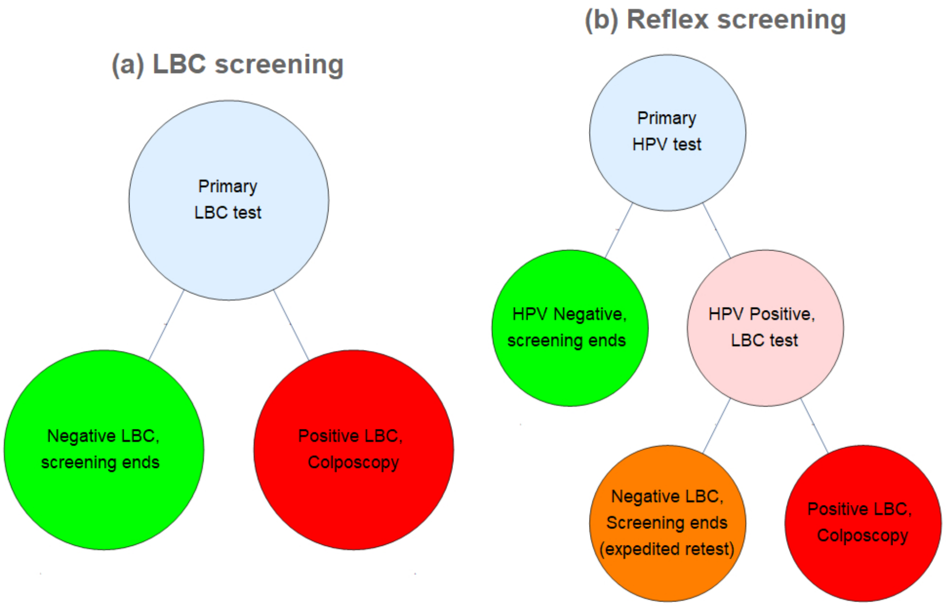

Clinical patient flow for (a) LBC screening and (b) HPV reflex screening. HPV positive, LBC negative results are frequently selected for an expedited retest outside of the normal screening interval

685x441mm (38 x 38 DPI)

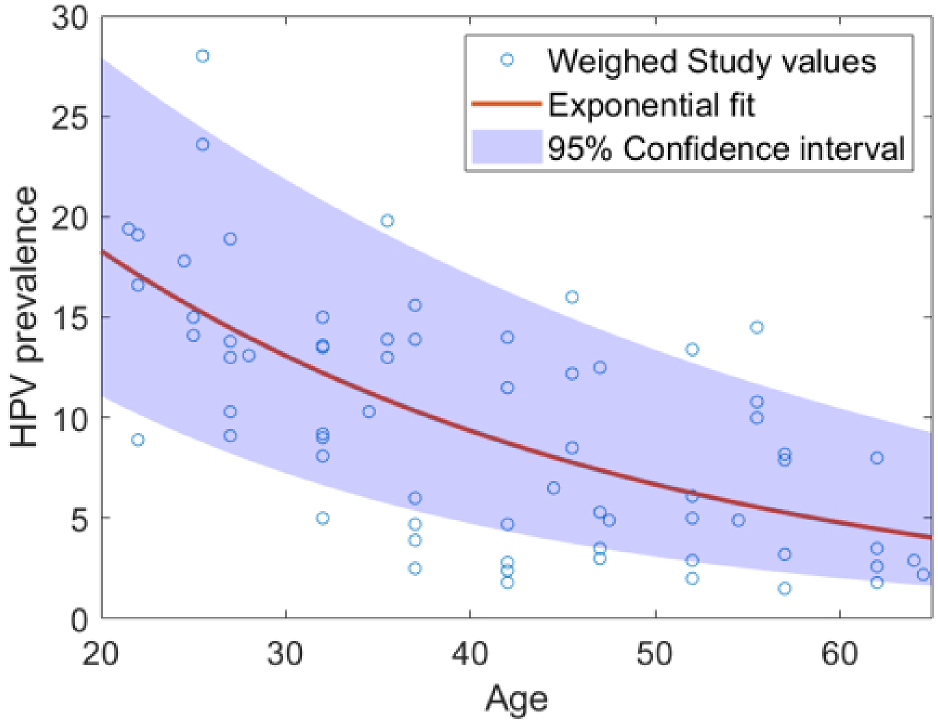

Figure 2 - Weighted fits from 12 European population studies with exponential fit and confidence intervals.

359x279mm (38 x 38 DPI)

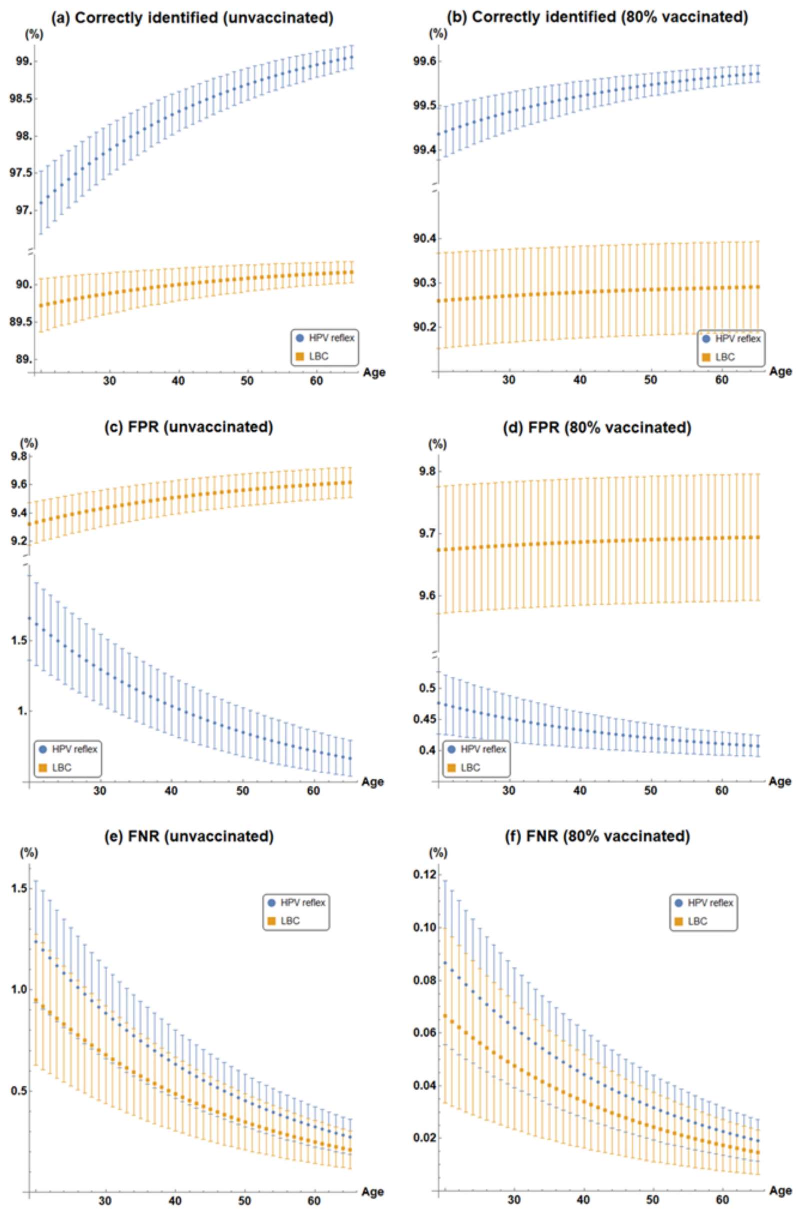

Figure 3: Performance of HPV-reflex and LBC screening modalities with age. Percentage correct results shown for (a) unvaccinated populations and (b) 80% vaccine uptake populations. False positive rates of both modalities with age shown for (c) unvaccinated populations and (d) 80% vaccine uptake populations. False negative rates depicted for (e) unvaccinated populations and (f) 80% vaccinated populations. Note the broken Y-axis in (a)-(d) inclusive

439x669mm (38 x 38 DPI)

Impact of Human Papillomavirus age-related prevalence and vaccination levels on interpretation of cervical screening modalities – a modelling study: Supplemental material

Author: David Robert Grimes[1,2], PhD

Addresses and affiliations:

1. School of Physical Sciences, Dublin City University, Glasnevin, Dublin, Ireland (davidrobert.grimes@dcu.ie)
2. Discipline of radiation therapy, Trinity College Dublin, Trinity Centre for Health Sciences, St. James's Hospital, Dublin 8, James's Street (davidrobert.grimes@tcd.ie)

40% Vaccination uptake simulation

Results as shown in figure 1.

For peer review only

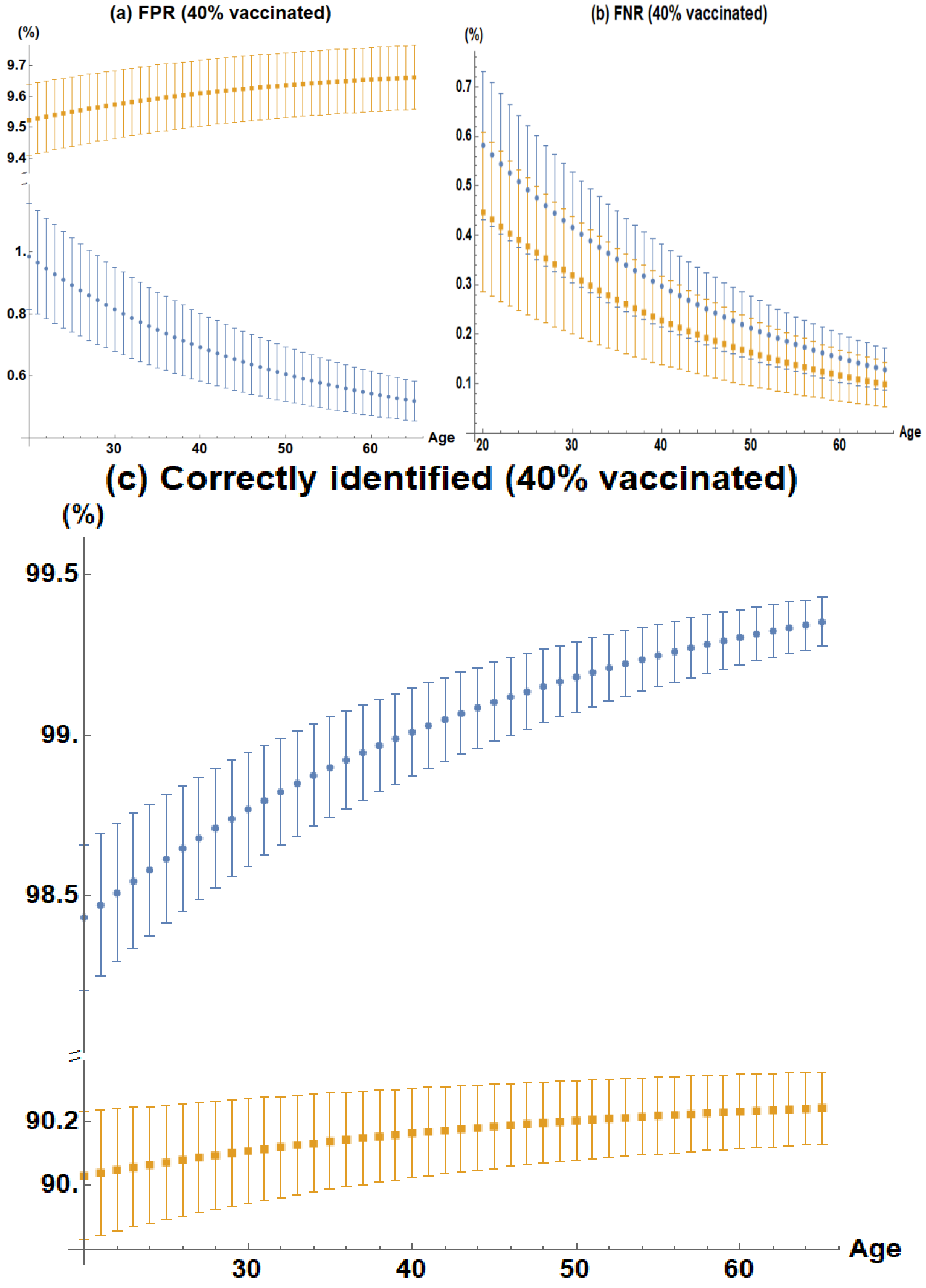

Figure 1 - 40% Vaccine uptake effect on (a) False positives (b) false negatives (c) correct identification

Age cohorts, sample size, and High risk HPV types

Study name	Number (n)	Age cohorts (N)	Hr- HPV types sampled
**Baay MF, Smits E, Tjalma WA, et al. Can cervical cancer screening be stopped at 50? The prevalence of HPV in elderly women.Int J Cancer 2004;108(2):258–61.**	1936	50-54, 55-59, 60-64, 65-69, 70+	16, 18, 31, 33, 35, 39, 45, 51, 52, 56, 58, 59, 66, 68.
**Vandenvelde C, Van Beers D. High-risk genital papillomaviruses and degree of dysplastic changes in the cervix: a prospective study by fast multiplex polymerase chain reaction in Belgium. J Med Virol 1993; 39(4):273**	323	20-30,31-40,41-50,51-60, 61-70	16,18,33
**Baay MF, Tjalma WA, Lambrechts HA, et al. Combined Pap and HPV testing in primary screening for cervical abnormalities:should HPV detection be delayed until age 35? Eur J Cancer 2005; 41(17):2704–8.**	2293	20-24, 25-29, 30-34, 35-39, 40-44, 45-50	16, 18, 31, 33, 35, 39, 45, 51, 52, 56, 58, 59, 66, 68
**Kjaer SK, van den Brule AJ, Bock JE, et al. Determinants for genital human papillomavirus (HPV) infection in 1000 randomly chosen young Danish women with normal Pap smear: are there different risk profiles for oncogenic and nononcogenic HPV types? Cancer Epidemiol Biomarkers Prev 1997;6(10):799–805**	956	20-23, 24-26, 27-29	16, 18, 31, 33, 35, 39, 45, 51, 52, 54, 56, 58, 59, 66, 68
**Boulanger JC, Sevestre H, Bauville E, et al. [Epidemiology of HPV infection]. Gynecol Obstet Fertil 2004;32(3):218–23**	3832	20-24, 25-29, 30-34, 35-39, 40-44,45-49, 50-54,55-59,60-64	16, 18, 31, 33, 35, 39, 45, 51, 52, 56, 58, 59 , 68
**Clavel C, Bory JP, Rihet S, et al. Comparative analysis of human papillomavirus detection by hybrid capture assay and routine cytologic screening to detect high-grade cervical lesions. Int J Cancer 1998; 75(4):525–8**	1028	14-20, 20-29, 30-39, 40-49, 50-59, 60-68	16, 18, 31, 33, 35, 45, 51, 52, 56
**Levert M, Clavel C, Graesslin O, et al. Human papillomavirus typing in routine cervical smears. Results from a series of 3778 patients]. Gynecol Obstet Fertil 2000;28(10):722–8**	3778	15-20, 21-30, 31-40, 41-50, 51-60	16, 18, 31, 33, 35, 39, 45, 51, 52, 56, 58, 59, 68
**Clavel C, Masure M, Bory JP, et al. Human papillomavirus testing in primary screening for the detection of high-grade cervical lesions: a study of 7932 women. Br J Cancer 2001;84(12):1616–23.**	7932	15-20, 21-30, 31-40, 41-50, 51-60	16, 18, 31, 33, 35, 39, 45, 51, 52, 56, 58, 59, 68
**Ronco G, Ghisetti V, Segnan N, et al. Prevalence of human papilloma- virus infection in women in Turin, Italy. Eur J Cancer 2005;41(2): 297–305.**	1013	25-29, 30-34, 35-39, 40-44, 45-49, 50-54, 55-59, 60-64	16, 18, 26, 31, 33, 34, 35, 39, 40, 42, 43, 44, 45, 51–59, 61, 66, 68, 70, 71, 72, 73, 81, 82, 83, 84, CP6108
**Jacobs MV, Walboomers JM, Snijders PJ, et al. Distribution of 37 mucosotropic HPV types in women with cytologically normal cervical**	3305	15-24, 25-29, 30-34, 35-39, 40-44, 45-49, 50-54, 55-	16, 18, 31, 33, 35, 39, 45, 51, 52, 56, 58, 59, 66, 68

For peer review only

**smears: the age-related patterns for high-risk and low-risk types. Int J Cancer 2000;87(2):221–7.**		59, 60-69	
**Cuzick J, Szarewski A, Terry G, et al. Human papillomavirus testing in primary cervical screening. Lancet 1995;345(8964):1533–6.**	2007	15-25, 25-29, 30-34, 35-39, 40-44, 45-49, 50-54, 55-69	16, 18, 31, 33
**Peto J, Gilham C, Deacon J, et al. Cervical HPV infection and neoplasia in a large population-based prospective study: the Manchester cohort. Br J Cancer 2004;91(5):942–53**	6128	15-19, 20-24, 25-29, 30-34, 35-39, 40-44, 45-49, 50-54, 55-69	16, 18, 26, 31, 33, 35, 39, 40, 45, 51, 52, 53, 54, 55, 56, 57, 58, 59, 66, 68, 73, 82, 83 84

For peer review only

TRIPOD Checklist: Prediction Model Development

Section/Topic	Item	Checklist Item	Page
**Title and abstract**			
Title	1	Identify the study as developing and/or validating a multivariable prediction model, the target population, and the outcome to be predicted.	2
Abstract	2	Provide a summary of objectives, study design, setting, participants, sample size, predictors, outcome, statistical analysis, results, and conclusions.	2-5
**Introduction**			
Background and objectives	3a	Explain the medical context (including whether diagnostic or prognostic) and rationale for developing or validating the multivariable prediction model, including references to existing models.	2-3
	3b	Specify the objectives, including whether the study describes the development or validation of the model or both.	2-3
**Methods**			
Source of data	4a	Describe the study design or source of data (e.g., randomized trial, cohort, or registry data), separately for the development and validation data sets, if applicable.	3-4
	4b	Specify the key study dates, including start of accrual; end of accrual; and, if applicable, end of follow-up.	N/A
Participants	5a	Specify key elements of the study setting (e.g., primary care, secondary care, general population) including number and location of centres.	3-4
	5b	Describe eligibility criteria for participants.	3-4
	5c	Give details of treatments received, if relevant.	N/A
Outcome	6a	Clearly define the outcome that is predicted by the prediction model, including how and when assessed.	4-5
	6b	Report any actions to blind assessment of the outcome to be predicted.	N/A
Predictors	7a	Clearly define all predictors used in developing or validating the multivariable prediction model, including how and when they were measured.	3-5
	7b	Report any actions to blind assessment of predictors for the outcome and other predictors.	NA
Sample size	8	Explain how the study size was arrived at.	NA
Missing data	9	Describe how missing data were handled (e.g., complete-case analysis, single imputation, multiple imputation) with details of any imputation method.	NA
Statistical analysis methods	10a	Describe how predictors were handled in the analyses.	3-5
	10b	Specify type of model, all model-building procedures (including any predictor selection), and method for internal validation.	2-3
	10d	Specify all measures used to assess model performance and, if relevant, to compare multiple models.	3-7
Risk groups	11	Provide details on how risk groups were created, if done.	2-3
**Results**			
Participants	13a	Describe the flow of participants through the study, including the number of participants with and without the outcome and, if applicable, a summary of the follow-up time. A diagram may be helpful.	NA
	13b	Describe the characteristics of the participants (basic demographics, clinical features, available predictors), including the number of participants with missing data for predictors and outcome.	2-3
Model development	14a	Specify the number of participants and outcome events in each analysis.	NA
	14b	If done, report the unadjusted association between each candidate predictor and outcome.	NA
Model specification	15a	Present the full prediction model to allow predictions for individuals (i.e., all regression coefficients, and model intercept or baseline survival at a given time point).	5
	15b	Explain how to the use the prediction model.	3-5
Model performance	16	Report performance measures (with CIs) for the prediction model.	5
**Discussion**			
Limitations	18	Discuss any limitations of the study (such as nonrepresentative sample, few events per predictor, missing data).	6-7
Interpretation	19b	Give an overall interpretation of the results, considering objectives, limitations, and results from similar studies, and other relevant evidence.	6-7
Implications	20	Discuss the potential clinical use of the model and implications for future research.	6-7
**Other information**			
Supplementary information	21	Provide information about the availability of supplementary resources, such as study protocol, Web calculator, and data sets.	6-7
Funding	22	Give the source of funding and the role of the funders for the present study.	7

We recommend using the TRIPOD Checklist in conjunction with the TRIPOD Explanation and Elaboration document.

For peer review only - http://bmjopen.bmj.com/site/about/guidelines.xhtml

BMJ Open

Impact of Human Papillomavirus age-related prevalence and vaccination levels on interpretation of cervical screening modalities – a modelling study

Journal:	*BMJ Open*
Manuscript ID	bmjopen-2023-078551.R1
Article Type:	Original research
Date Submitted by the Author:	03-Jan-2024
Complete List of Authors:	Grimes, David; University of Oxford, & Queens University Belfast
**Primary Subject Heading**:	Public health
Secondary Subject Heading:	Sexual health, Public health
Keywords:	Mass Screening, PUBLIC HEALTH, Public health < INFECTIOUS DISEASES, Sexually Transmitted Disease

SCHOLARONE™
Manuscripts

I, the Submitting Author has the right to grant and does grant on behalf of all authors of the Work (as defined in the below author licence), an exclusive licence and/or a non-exclusive licence for contributions from authors who are: i) UK Crown employees; ii) where BMJ has agreed a CC-BY licence shall apply, and/or iii) in accordance with the terms applicable for US Federal Government officers or employees acting as part of their official duties; on a worldwide, perpetual, irrevocable, royalty-free basis to BMJ Publishing Group Ltd ("BMJ") its licensees and where the relevant Journal is co-owned by BMJ to the co-owners of the Journal, to publish the Work in this journal and any other BMJ products and to exploit all rights, as set out in our licence.

The Submitting Author accepts and understands that any supply made under these terms is made by BMJ to the Submitting Author unless you are acting as an employee on behalf of your employer or a postgraduate student of an affiliated institution which is paying any applicable article publishing charge ("APC") for Open Access articles. Where the Submitting Author wishes to make the Work available on an Open Access basis (and intends to pay the relevant APC), the terms of reuse of such Open Access shall be governed by a Creative Commons licence – details of these licences and which Creative Commons licence will apply to this Work are set out in our licence referred to above.

Other than as permitted in any relevant BMJ Author's Self Archiving Policies, I confirm this Work has not been accepted for publication elsewhere, is not being considered for publication elsewhere and does not duplicate material already published. I confirm all authors consent to publication of this Work and authorise the granting of this licence.

For peer review only

Impact of Human Papillomavirus age-related prevalence and vaccination levels on interpretation of cervical screening modalities – a modelling study

Author: David Robert Grimes[1,2], PhD

Addresses and affiliations:

1. Discipline of radiation therapy, Trinity College Dublin, Trinity Centre for Health Sciences, St. James's Hospital, Dublin 8, James's Street (davidrobert.grimes@tcd.ie)
2. School of Physical Sciences, Dublin City University, Glasnevin, Dublin, Ireland (davidrobert.grimes@dcu.ie)

Word count: 2500

Figure count: 3

Table count: 2

For peer review only

Impact of Human Papillomavirus age-related prevalence and vaccination levels on interpretation of cervical screening modalities – a modelling study

David Robert Grimes, PhD –davidrobert.grimes@tcd.ie

Abstract

Objective: Cervical screening is a life-saving intervention which reduces the incidence of and mortality from cervical cancer in the population. Human Papillomavirus (HPV) based screening modalities hold unique promise in improving screening accuracy. HPV prevalence varies markedly by age, as does resultant cervical intra-epithelial neoplasia (CIN), with higher rates recorded in younger women. With the advent of effective vaccination for HPV drastically reducing prevalence of both HPV and CIN, it is critical to model how the accuracy of different screening approaches varies with age-cohort and vaccination status. This work establishes a model for age-specific prevalence of HPV factoring in vaccine coverage, and predicts how the accuracy of common screening modalities is affected by age profile and vaccine uptake.

Design: Modelling study of HPV infection rates by age, ascertained from European cohorts prior to the introduction of vaccination. Reductions in HPV due to vaccination were estimated from the bounds predicted from multiple modelling studies, yielding a model for age-varying HPV and CIN grades 2 and above (CIN2+) prevalence.

Setting: Performance of both conventional Liquid-base cytology (LBC) screening and HPV screening with LBC reflex (HPV-reflex) were estimated under different simulated age cohorts and vaccination levels.

Participants: Simulated populations of varying age and vaccination status

Results: HPV-reflex modalities consistently result in much lower incidence of false positives than LBC testing, with an accuracy that improves even as HPV and CIN2+ rates decline.

Conclusions: HPV-reflex tests outperform LBC tests across all age profiles, resulting in greater test accuracy. This improvement is especially pronounced as HPV infection rates fall and suggests HPV-reflex modalities are robust to future changes in the epidemiology of HPV.

Strengths and weaknesses

- Extends modelling approaches to screening outcomes by incorporating the projected HPV prevalence and vaccine uptake levels of age-varying cohort.
- Provides a novel methodology for ascertaining how screening modalities will perform under likely future scenarios.
- HPV prevalence estimates are limited by assumptions efficacy and uptake of vaccination, and this can be varied with the model presented.

For peer review only

Introduction

Cervical cancer screening is a powerful life-saving intervention, associated with significant reductions in the mortality and cancer burden [1-3]. For squamous cell carcinoma in women older than 25 years, national screening paradigms have resulted in an estimated 80% reduction in mortality. Human papillomavirus (HPV) is responsible for most of these cancers, and the advent of vaccination against its oncogenic strains has already had transformative impact on both rates of HPV and cervical intra-epithelial neoplasia (CIN) [2] .

Alongside conventional Liquid based cytology (LBC) modalities for screening for CIN grades 2 and higher (CIN2+), the evolution of screening modalities to include HPV-reflex method which triage cases by HPV infection status have shown great promise [4-6]. This promise however requires careful interpretation - HPV testing is often deemed to have higher sensitivity than conventional LBC approaches, but there are nuances which demand careful consideration. The sensitivity of HPV tests is not a direct proxy to LBC sensitivity, as they measure different things. While LBC tests directly measure the presence of CIN2+, HPV tests themselves only detect the presence of high-risk strains of HPV. In younger women especially, there is high transient incidence of HPV, and using HPV infection alone as a screening modality would result in an unacceptably high false detection rate of CIN2+. False positive results not only induce anxiety in patients but also lead to potential overtreatment, with the potential to overwhelm colposcopy services unless these are robustly designed.

Accordingly, HPV triage approaches are typically implemented, where patients testing positive for high-risk HPV receive a reflex LBC, while those testing negative do not. A recent analysis [3] demonstrated that this reflex approach has in fact a slightly lower effective sensitivity for CIN2+ (0.68, 95% Confidence interval: 0.60-0.75) than LBC testing alone (0.76, 95% Confidence interval: 0.67-0.83) in a typical population, though this seeming disadvantage is typically mitigated by expedited retesting of HPV positive results before the next screening cycle. The chief advantage of HPV-reflex tests (or LBC tests with HPV reflex) is that they hugely decrease the false positive rate, with a reflex test yielding 9.6 false positives per 1000 women (95% Confidence interval: 9.4-9.8) compared to LBC testing with 95.1 false positives per 1000 women (95% Confidence interval: 93.7- 97.0), decreasing false positive rates by approximately ten-fold and reducing overtreatment. Simplified clinical patient flow for both LBC and HPV reflex modalities are illustrated in figure 1.

Yet to date, this work has been largely considered average HPV infection rates and CIN2+ prevalence averaged across entire populations. HPV infection rates however differ by both age cohort [7] and vaccination status [8]. Younger women tend to have higher rates of both HPV infection, and CIN2+. This in turn has implications for current and future screening interpretations and planning with differing cohorts. CIN2+ prevalence is directly proportional to HPV infection [9] and will change with as infection rates change either due to aging cohorts or increased vaccine uptake. A simple dynamic model to account for this would allow accurate determination of screening performance under realistic scenarios and facilitate the planning of future screening modalities. This work accordingly establishes a model to account HPV infection rates, and CIN2+ prevalence with age cohorts and vaccine uptake, ascertaining test accuracy under all possible future scenarios.

Figure 1 - Clinical patient flow for (a) LBC screening and (b) HPV reflex screening. HPV positive, LBC negative results are frequently selected for an expedited retest outside of the normal screening interval

Methods

Modelling varying prevalence of HPV and CIN2+

For peer review only

HPV infection rates vary markedly by country and cohort, and even detection methodology. The evidence to date strongly suggests that HPV infection peaks before 30, though slightly different trends emerge in various data sets. For the purposes of this investigation, analysis was restricted to sets prior to widespread coverage of the HPV vaccine. On current estimates from USA and European data, we expect a global average of approximately 8.4% of the total population to have active HPV-infection at the time of screening [3]. This figure pertains to the total prevalence across the population, and the caveats are elucidated in the discussion.

For this work, it was crucial to further stratify this figure by age cohort. To derive an age-specific HPV prevalence rate, we considered the 2008 review by Smith et al [7] of the literature on HPV infection rates throughout the world. This review included various data sources, including these stemming from sexual infection clinics or specific high-risk populations. As these results are likely to produce high (or in some cases low) outliers which can skew analysis, we confined ourselves only to the subset of studies solely from general European screening programmes. Furthermore, this analysis only considered studies with some degree of age stratification. This resulted in 12 applicable studies [10-21] . These studies were undertaken in various cities throughout Belgium, Denmark, France, Italy, The Netherlands, and the United Kingdom between 1992 and 2005, with samples ranging from 323 women to 7932 women (mean: 2878 women). Data from these studies was then taken and weighted according to the number of study participants and smoothed, so that an age-varying phenomenological model describing HPV prevalence at age in years *a* could be ascertained with 95% confidence intervals, denoted $h_u(a)$. Details of these studies, participants, settings, and HPV subtypes tested for are given in the supplementary material.

Crucially, these estimates stem from before the advent of HPV vaccination, and accordingly map to a scenario of 0% vaccine coverage. This is a useful baseline upon which we can model the impact of vaccination on test performance. The impact of HPV vaccination on prevalence rates was derived from 29 previously published modelling studies [8], yielding an estimate for *V*, the reduction factor for HPV prevalence from given level of vaccination, as previously published [3,8]. The time-varying HPV prevalence for a given level of vaccination is thus $h(a) = (1 - V)h_u(a)$, with the mathematical justification for this simplification given in the supporting mathematical appendix. For brevity, results in this work consider the impact of vaccine rates of 0% (completely unvaccinated cohorts) and 80% (highly vaccinated cohorts) with mid-level vaccination rates of 40% given in the supplementary material. The mean value for $h(a)$ across the entire span of screening ages is given by

$$\bar{\mathrm{h}} = \frac{1}{\mathrm{a_e} - \mathrm{a_s}} \int_{a_s}^{\mathrm{a_e}} \mathrm{h}(a)\mathrm{da}\,.$$

Taking the upper bound of screening ages to be $\mathrm{a_e} = 65$ years and the lower bound to be $\mathrm{a_s} = 20$ years, the mean HPV prevalence in the cohort can be ascertained from the form of the phenomenological function. The CIN2/3 prevalence is proportional to the rates of HPV infection, with a justifying argument for this given in the mathematical supplementary material. It follows then that a simple scaling function should be predictive of prevalence rates for CIN2+. If the known population average of CIN2+ is $\bar{\mathrm{p}}$, then age / vaccine varying CIN2+ prevalence is accordingly given by

$$p(a) = \left(\frac{\bar{p}}{\bar{h}}\right) h(a).$$

Interpretation of screening results with varying HPV prevalence and vaccine uptake.

For peer review only - http://bmjopen.bmj.com/site/about/guidelines.xhtml

With age and vaccination specific CIN2/3 prevalence known, one can explicitly calculate false positive rates, false negative rates, and over-all correct identifications using previously published methods. For Liquid based cytology (LBC), the false positive (fp_l), false negative (fn_l), and over-all correct identification rates (t_l) are related to CIN2/3 prevalence and the sensitivity (s_{nl}) and specificity (s_{pl}) of LBC testing, given respectively by the relationships

$$fp_l(a) = (1 - p(a))(1 - s_{pl})$$

$$fn_l(a) = p(a)(1 - s_{nl})$$

$$t_l(a) = 1 - (f_{pl}(a) + f_{nl}(a)).$$

For HPV reflex testing, false positive rates (fp_r), false negative rates (fn_r),, and over-all correct identification rate (t_r) depend additionally on HPV prevalence, the sensitivity of HPV testing (s_{nh}), the specificity of HPV testing (s_{ph}) and the fraction of CIN2+ cases associated with detectable HPV, v. These equations are derived from those previously published[3], and are respectively

$$fp_r(a) = (1 - s_{pl})(s_{nh}(h(a) - p(a)v) + (1 - s_{ph})(1 - h(a) - p(a)(1 - v)))$$

$$fn_r(a) = vp(a)(1 - s_{nl}s_{nh}) + p(a)(1 - v)(1 - s_{nl}(1 - s_{ph}))$$

$$t_r(a) = 1 - (f_{pl}(a) + f_{nl}(a)).$$

Literature values used for all modelling simulations are given in table 1.

Table 1: Simulation values and source

Parameter	Values (95% CI where available)
Average prevalence of CIN2/3 in full population ($\bar{p}$)	0.02[6]
Sensitivity of LBC test to CIN2+ (s_{nl})	0.76 ± 0.04 [6]
Specificity of LBC test to CIN2+ (s_{pl})	0.903 ± 0.001 [6]
Sensitivity of HPV test to High-Risk HPV (s_{nh})	$0.947\ (-)$ [3]
Specificity of HPV test to High-Risk HPV (s_{ph})	0.96 ± 0.001 [6]
CIN2/3 attributable to testable hr-HPV (v)	$0.95(-)$ [3]
Reduction in HPV at 40% vaccine coverage (V_{40})	0.53 ± 0.06 [3,8]
Reduction in HPV at 80% vaccine coverage (V_{80})	0.93 ± 0.026 [3,8]

For peer review only

Patient and Public Involvement

None. It was not appropriate or possible to involve patients or the public in the design, or conduct, or reporting, or dissemination plans of this research.

Results

Modelling varying prevalence of HPV and CIN2+

Age-related HPV prevalence from 12 included studies were found to approximately obey an exponential decay type-model from ages 20-65 of the form $A\exp(-\beta t)$ as illustrated in figure 1 ($A = 35.81 \pm 4.91$, $\beta = 0.0336 \pm 0.0045\ yr^{-1}$, 95% confidence interval). This envelope with weighed study data points are shown in figure 1. The estimate for mean HPV infection rate in unvaccinated populations across all age ranges was $\bar{h} = 9.4 \pm 2.3\,\%$, in good agreement with literature estimate of 8.4%, and with population wide European estimates of 9.8% [22] (95% Confidence Interval: 9.2%-10.0%) and from the ATHENA study [23] (10.5%, 95% Confidence Interval: 10.3%-10.7%) . CIN2+ rates by age cohort from inferred from this as per the model outline.

Figure 2 - Weighted fits from 12 European population studies with exponential fit and confidence intervals.

Interpretation of screening results with varying HPV prevalence and vaccine uptake

Figure 2 depicts how the accuracy of LBC and HPV-reflex modalities (in terms of true results, false positives, and false negatives) change with age in both unvaccinated and 80% vaccinated populations. HPV reflex modalities consistently outperform LBC modalities for all age cohorts and vaccine uptake levels. Table 2 depicts a selection of results for varying age profiles and vaccine uptake, with additional Simulation results for 40% vaccine coverage are given in the supplementary material.

Figure 3: Performance of HPV-reflex and LBC screening modalities with age. Percentage correct results shown for (a) unvaccinated populations and (b) 80% vaccine uptake populations. False positive rates of both modalities with age shown for (c) unvaccinated populations and (d) 80% vaccine uptake populations. False negative rates depicted for (e) unvaccinated populations and (f) 80% vaccinated populations. Note the broken Y-axis in (a)-(d) inclusive

Table 2: Modelled test performance at specific ages and vaccination levels

	LBC			HPV reflex		
**Age profile**	***False Negative %***	***False Positive %***	***Correct Result %***	***False Negative %***	***False Positive %***	***Correct Result %***
Age 25						
**0% Vaccinated**	0.8 ± 0.28	9.38 ± 0.14	89.1 ± 0.31	1.05 ± 0.26	1.46 ± 0.27	97.50 ± 0.40
**40% Vaccinated**	0.38 ± 0.14	9.55 ± 0.11	90.07 ± 0.18	0.49 ± 0.13	1.61 ± 0.26	97.89 ± 0.29
**80% Vaccinated**	0.06 ± 0.03	9.68 ± 0.10	90.27 ± 0.11	0.07 ± 0.03	1.73 ± 0.26	98.20 ± 0.26
Age 35						
**0% Vaccinated**	0.57 ± 0.21	9.47 ± 0.12	89.95 ± 0.24	0.75 ± 0.20	1.16 ± 0.23	98.10 ± 0.30

**40% Vaccinated**	0.27 ± 0.10	9.59 ± 0.11	90.14 ± 0.15	0.35 ± 0.10	1.26 ± 0.22	98.38 ± 0.24
**80% Vaccinated**	0.04 ± 0.02	9.68 ± 0.10	90.28 ± 0.10	0.05 ± 0.02	1.35 ± 0.22	98.60 ± 0.22
Age 45						
**0% Vaccinated**	0.41 ± 0.16	9.54 ± 0.12	90.05 ± 0.20	0.53 ± 0.15	0.94 ± 0.19	98.53 ± 0.24
**40% Vaccinated**	0.19 ± 0.08	9.62 ± 0.11	90.18 ± 0.11	0.25 ± 0.07	1.01 ± 0.18	98.73 ± 0.20
**80% Vaccinated**	0.03 ± 0.02	9.69 ± 0.10	90.28 ± 0.10	0.04 ± 0.01	1.07 ± 0.18	98.89 ± 0.18
Age 55						
**0% Vaccinated**	0.29 ± 0.12	9.58 ± 0.11	90.12 ± 0.16	0.38 ± 0.11	0.78 ± 0.16	98.84 ± 0.19
**40% Vaccinated**	0.14 ± 0.06	9.65 ± 0.10	90.22 ± 0.12	0.18 ± 0.06	0.84 ± 0.15	98.99 ± 0.16
**80% Vaccinated**	0.02 ± 0.01	9.69 ± 0.10	90.29 ± 0.10	0.03 ± 0.01	0.88 ± 0.15	99.10 ± 0.10

For peer review only

Discussion

In all simulated cases with realistic parameters, HPV-reflex testing correctly identifies a higher proportion of cases ($97.1 \pm 0.4\%$ to $99.6 \pm 0.2\%$) relative to LBC testing for the same scenarios ($89.7 \pm 0.4\%$ to $90.3 \pm 0.1\%$). This is seen clearly in Figure 3(a) and (b), and detailed in table 2. The superior performance of HPV-reflex manifests chiefly due to markedly lower false positive rates. In all scenarios, LBC false positive rates exceed HPV-reflex values for all scenarios by a factor of 5.6 to 23.8. This analysis focused on a comparison of HPV-reflex to LBC testing. It did not explicitly consider co-testing, where both LBC and HPV screening are performed and a positive on either leads to colposcopy, as this modality leads to unacceptably high false positives results, although it is possible in principle to extend the analysis here to consider how this scenario would vary by age cohort and vaccination status.

This difference is particularly pronounced in vaccinated cohorts, as false positives decline markedly with lower HPV prevalence in HPV-reflex testing. By contrast, false positive rates slightly increase in vaccinated cohorts with LBC testing, as seen in figure 3(c)-(d). This Is expected, as the limit of LBC false positive rate as CIN2+ prevalence approaches zero is simply $1 - s_{pl}$, yielding a theoretical maximum possible false positive rate for LBC of approximately 9.7%. By contrast, false positives decline with reflex with HPV, resulting in a theoretical minimum false positive rate of $(1 - s_{pl})(1 - s_{ph})$, or a minimum value of approximately 0.39%.

For both LBC and reflex testing, false negatives decline with decreasing HPV prevalence. LBC testing does have a slightly lower false negative rate than HPV-reflex testing, but this is marginal and overlaps with the performance envelope for HPV-reflex testing. In principle, LBC testing is more sensitive to instances of CIN2+ than reflex screening, but it results in far more false positives and unnecessary referrals to colposcopy. As HPV detection sensitivity is so high, expedited retesting can be employed when a patient tests positive for a high-risk strain of HPV, and negative on LBC. This is likely to more than compensate for the marginally higher false negative rate of reflex testing, while preserving the crucial benefit and over-all accuracy. Expedited retesting is already implemented for HPV positive results in several screening programmes, including the Irish National screening programme CervicalCheck [24] . where HPV positive but cytology negative patients are recalled for repeat screening after one year rather than three or five. Such approaches maintain the benefits of HPV-reflex without the potential drawback of lower net-sensitivity.

One limitation of the current study was the assumption that CIN2+ prevalence corresponds linearly to HPV prevalence, yielding an approximately constant proportion with age cohort. This is a reasonable

assumption as justified in the mathematical appendix, but there are several important caveats that need to be considered. Firstly, the proportion estimated in this work pivots on how well the populations in the HPV and CIN2+ datasets correspond to one another. While these data sets were chosen to avoid the potentially skewing influence of elective sexually transmitted disease clinic settings, there may be other confounding reasons why they are not representative, in which case estimates of $p(a)$ might be biased.

In addition, the proportionality itself might vary with time for biological reasons. While younger women for example might have a higher rate of HPV, it is conceivable that they have better mechanisms of clearance, and so proportionally might have a lower fraction of CIN2+ compared to older cohorts. In the absence of clear data, this is of course speculative but the existence of any such age-specific variation in clearance rate would potentially impact the estimates in this work.

It is worth briefly discussing the mean HPV infection rate in unvaccinated populations across all age ranges, calculated as $\bar{h} = 9.4 \pm 2.3$ % in this work. This figure is in good agreement with literature estimate of 8.4% as described, and with population wide European estimates of 9.8% [22] (95% Confidence Interval: 9.2%-10.0%) and more recent results from the ATHENA study [23]. Estimates in literature however can be someway complicated by how sampling conducted – samples drawn from opportunistic testing or from specific environments like sexual health clinics, or samples skewed heavily towards youth may tend to over-estimate prevalence. Bruni et al [22], for example, in their meta-analysis reported a prevalence of 14.2% (ranging from 8.8% in Southern Europe to 21.4% in Eastern Europe.

These estimates are readily confounded by the age and selection bias of subjects in the underlying studies (for example, studies disproportionately consisting of younger women or opportunistic testing), hence the disparity with the reported population prevalence even in the same work. In this model, $\bar{h}$ is data derived, but we can also force a higher value into the simulation to examine consistency ever with much higher prevalence. The supplementary material contains a simulation assigning $\bar{h} = 15\%$ to investigate this, and demonstrates that even this much higher contrived estimate has only negligible impact on false positive and negative rates.

It is important to note that this analysis did not consider the impacts of follow-up between screening rounds, nor the effects intervals between screening rounds, yielding instead an age-specific point estimate of false positive and negative rates in isolation. The cumulative false positive and negative rate over multiple screening rounds will vary depending on the intervals between screening, the rate of HPV clearance, and the modalities employed. This is an involved question beyond the scope of this work, and the impacts of repeated screening on false negative and positive rates require dedicated future analysis to answer fully.

In consequence, HPV-reflex testing is calculated to be more robust to changing age and vaccine status than LBC testing and will increase further in accuracy as HPV infection decreases due to vaccination. This is an important consideration for the planning and interpretation of future screening and colposcopy services, which need to be robust for both age variation of HPV infection, and the eventuality where HPV infection is virtually eradicated due to increased uptake of the HPV vaccine. This is especially critical considering the World Health Organisations' cervical cancer elimination strategy 90-70-90 targets. These proposed measures [25], to be put in place across nations worldwide by 2030 to eliminate cervical cancer, comprise 90% of girls vaccinated against HPV by age 15, 70% of women screened by age 35, and 90% of people identified with cervical disease treated. The results of this analysis strongly suggest that HPV-reflex screening approaches are robust to this changing

For peer review only

epidemiology of HPV infection, and will continue to perform strongly even as HPV infections fall with increasing vaccine uptake.

Competing interests statement

Dr Grimes reported that he has written articles on both screening and HPV vaccination for popular audiences, receiving no industry funding for these articles. He also reports that he has consulted for public health bodies and charities in Europe on screening. He also reported receiving paid royalties for a popular science book and receiving fees and honoraria for contributed articles, fact-checking, public talks, and media appearances.

Ethical approval statement

This work involved no patient or personal data and only used publicly available and previously published prevalence data. Accordingly, no ethical approval was required for this work.

Funding statement

This work was funded by grant number 214461/Z/18/Z from the Wellcome Trust. The funder had no role in the design and conduct of the study; collection, management, analysis, and interpretation of the data; preparation, review, or approval of the manuscript; and decision to submit the manuscript for publication.

Data sharing statement

Source code for the simulations herein are hosted online at https://github.com/drg85/CervScreenAgeVax

Authors' contributions

DRG conceived the model, analysed the published data, performed the analysis, reported the results and wrote the manuscript.

References

1. Peto, J., Gilham, C., Fletcher, O., & Matthews, F. E. (2004). The cervical cancer epidemic that screening has prevented in the UK. The Lancet, 364(9430), 249-256.
2. Hall, M. T., Simms, K. T., Lew, J. B., Smith, M. A., Brotherton, J. M., Saville, M., ... & Canfell, K. (2019). The projected timeframe until cervical cancer elimination in Australia: a modelling study. The Lancet Public Health, 4(1), e19-e27.
3. Grimes, D. R., Corry, E. M., Malagón, T., O'Riain, C., Franco, E. L., & Brennan, D. J. (2021). Modeling cervical cancer screening strategies with varying levels of human papillomavirus vaccination. JAMA network open, 4(6), e2115321-e2115321.
4. Franco EL, Mahmud SM, Tota J, Ferenczy A, Coutlée F. The expected impact of HPV vaccination on the accuracy of cervical cancer screening: the need for a paradigm change. Arch Med Res. 2009;40(6):478-485
5. Ronco G, Dillner J, Elfström KM, et al; International HPV screening working group. Efficacy of HPV-based screening for prevention of invasive cervical cancer: follow-up of four European randomised controlled trials. Lancet. 2014;383(9916):524-532. doi:10.1016/S0140-6736(13)62218-7

For peer review only

6. Koliopoulos G, Nyaga VN, Santesso N, et al. Cytology versus HPV testing for cervical cancer screening in the general population. Cochrane Database Syst Rev. 2017;8:CD008587. doi:10.1002/14651858.CD008587.pub2
7. Smith, J. S., Melendy, A., Rana, R. K., & Pimenta, J. M. (2008). Age-specific prevalence of infection with human papillomavirus in females: a global review. Journal of Adolescent Health, 43(4), S5-e1.
8. Brisson, M., Bénard, É., Drolet, M., Bogaards, J. A., Baussano, I., Vänskä, S., ... & Walsh, C. (2016). Population-level impact, herd immunity, and elimination after human papillomavirus vaccination: a systematic review and meta-analysis of predictions from transmission-dynamic models. The Lancet Public Health, 1(1), e8-e17.
9. Ting J, Kruzikas DT, Smith JS. A global review of age-specific and overall prevalence of cervical lesions. Int J Gynecol Cancer. 2010 Oct;20(7):1244-9. doi: 10.1111/igc.0b013e3181f16c5f. PMID: 21495248.
10. Baay MF, Smits E, Tjalma WA, et al. Can cervical cancer screening be stopped at 50? The prevalence of HPV in elderly women.Int J Cancer 2004;108(2):258–61.
11. Vandenvelde C, Van Beers D. High-risk genital papillomaviruses and degree of dysplastic changes in the cervix: a prospective study by fast multiplex polymerase chain reaction in Belgium. J Med Virol 1993; 39(4):273
12. Baay MF, Tjalma WA, Lambrechts HA, et al. Combined Pap and HPV testing in primary screening for cervical abnormalities:should HPV detection be delayed until age 35? Eur J Cancer 2005; 41(17):2704–8.
13. Kjaer SK, van den Brule AJ, Bock JE, et al. Determinants for genital human papillomavirus (HPV) infection in 1000 randomly chosen young Danish women with normal Pap smear: are there different risk profiles for oncogenic and nononcogenic HPV types? Cancer Epidemiol Biomarkers Prev 1997;6(10):799–805
14. Boulanger JC, Sevestre H, Bauville E, et al. [Epidemiology of HPV infection]. Gynecol Obstet Fertil 2004;32(3):218–23
15. Clavel C, Bory JP, Rihet S, et al. Comparative analysis of human papillomavirus detection by hybrid capture assay and routine cytologic screening to detect high-grade cervical lesions. Int J Cancer 1998; 75(4):525–8
16. Levert M, Clavel C, Graesslin O, et al. Human papillomavirus typing in routine cervical smears. Results from a series of 3778 patients]. Gynecol Obstet Fertil 2000;28(10):722–8
17. Clavel C, Masure M, Bory JP, et al. Human papillomavirus testing in primary screening for the detection of high-grade cervical lesions: a study of 7932 women. Br J Cancer 2001;84(12):1616–23.
18. Ronco G, Ghisetti V, Segnan N, et al. Prevalence of human papilloma- virus infection in women in Turin, Italy. Eur J Cancer 2005;41(2): 297–305.
19. Jacobs MV, Walboomers JM, Snijders PJ, et al. Distribution of 37 mucosotropic HPV types in women with cytologically normal cervical smears: the age-related patterns for high-risk and low-risk types. Int J Cancer 2000;87(2):221–7.
20. Cuzick J, Szarewski A, Terry G, et al. Human papillomavirus testing in primary cervical screening. Lancet 1995;345(8964):1533–6.
21. Peto J, Gilham C, Deacon J, et al. Cervical HPV infection and neoplasia in a large population-based prospective study: the Manchester cohort. Br J Cancer 2004;91(5):942–53
22. Bruni, L., Diaz, M., Castellsagué, M., Ferrer, E., Bosch, F. X., & de Sanjosé, S. (2010). Cervical human papillomavirus prevalence in 5 continents: meta-analysis of 1 million women with normal cytological findings. Journal of Infectious Diseases, 202(12), 1789-1799.

For peer review only

23. Wright, T. C., Stoler, M. H., Behrens, C. M., Sharma, A., Zhang, G., & Wright, T. L. (2015). Primary cervical cancer screening with human papillomavirus: end of study results from the ATHENA study using HPV as the first-line screening test. Gynecologic oncology, 136(2), 189-197.
24. CervicalCheck National Screening Programme, Health Service Executive Ireland. Cervical Screening results and recommendations table CS/PUB/LAB-2 Rev. 12 (2021) – Available online from https://www.cervicalcheck.ie/_fileupload/Health-professionals/CervicalScreeningResultsandRecommendations.pdf
25. Wilailak, S., Kengsakul, M., & Kehoe, S. (2021). Worldwide initiatives to eliminate cervical cancer. International Journal of Gynecology & Obstetrics, 155, 102-106.

For peer review only

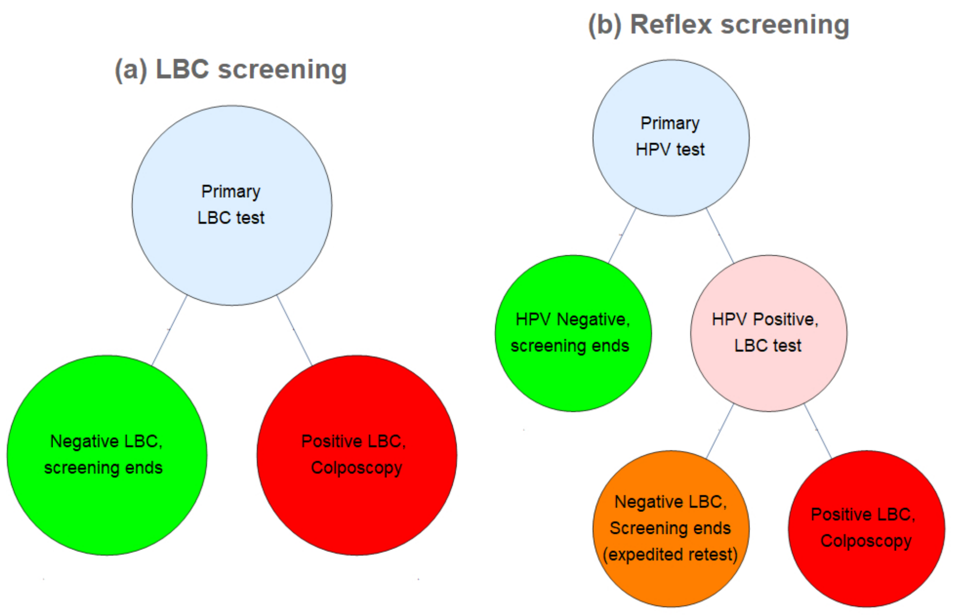

Clinical patient flow for (a) LBC screening and (b) HPV reflex screening. HPV positive, LBC negative results are frequently selected for an expedited retest outside of the normal screening interval

685x441mm (38 x 38 DPI)

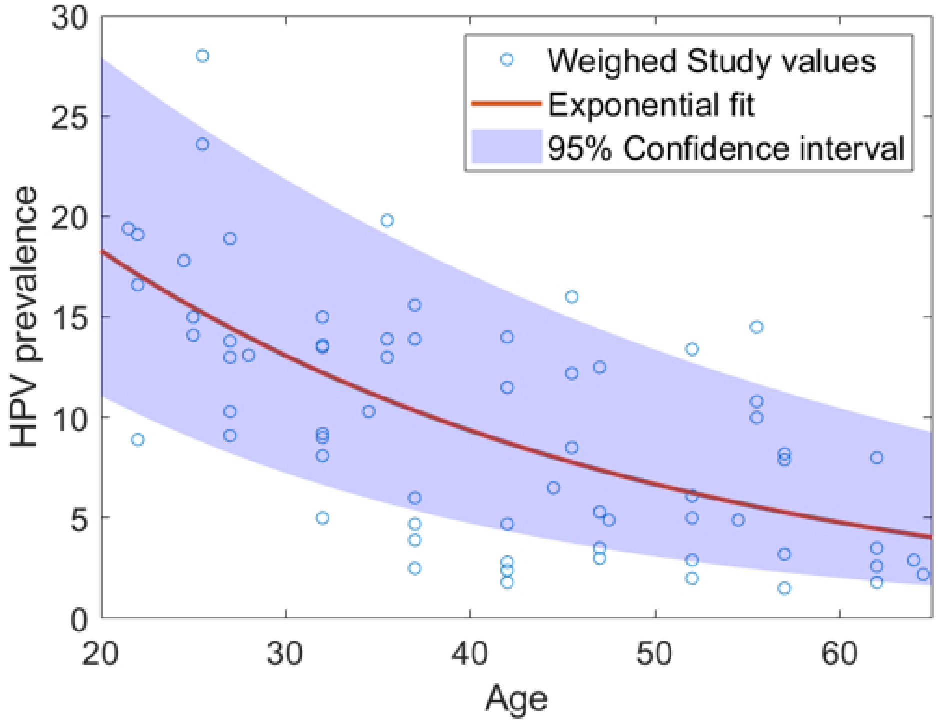

Figure 2 - Weighted fits from 12 European population studies with exponential fit and confidence intervals.

359x279mm (38 x 38 DPI)

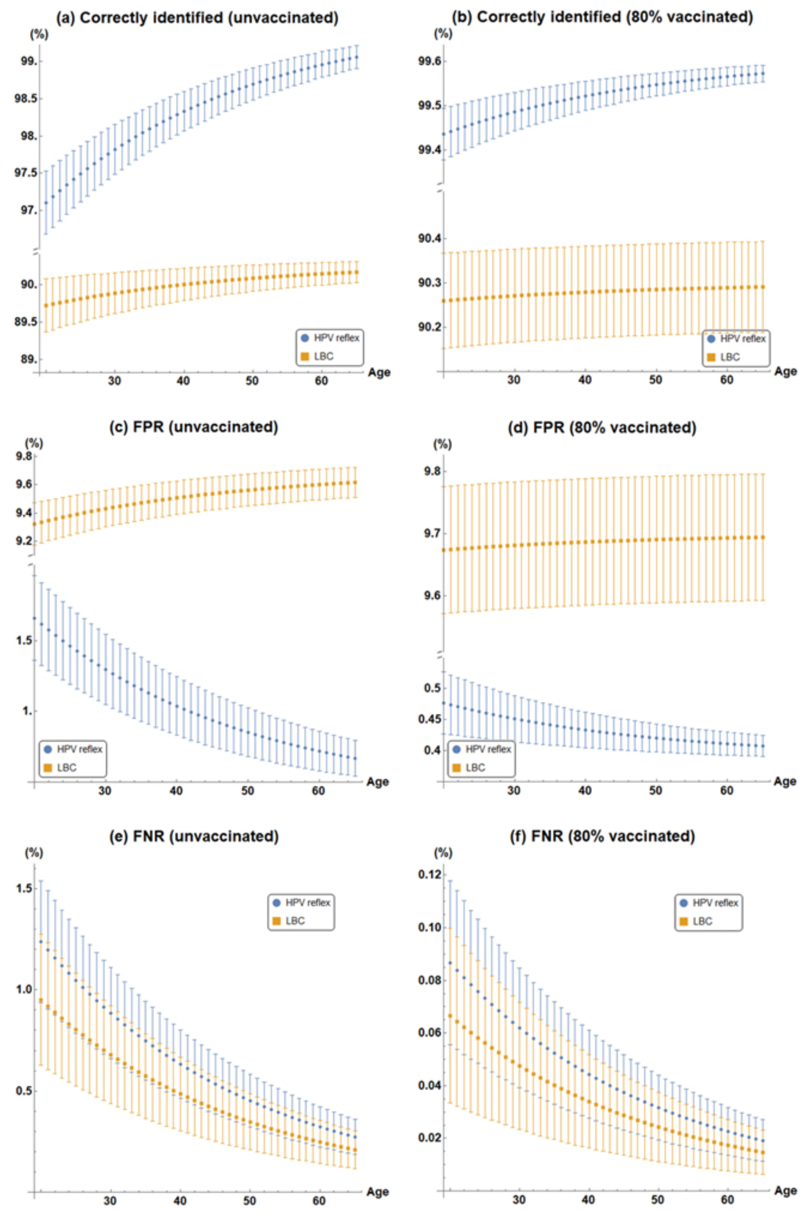

Figure 3: Performance of HPV-reflex and LBC screening modalities with age. Percentage correct results shown for (a) unvaccinated populations and (b) 80% vaccine uptake populations. False positive rates of both modalities with age shown for (c) unvaccinated populations and (d) 80% vaccine uptake populations. False negative rates depicted for (e) unvaccinated populations and (f) 80% vaccinated populations. Note the broken Y-axis in (a)-(d) inclusive

439x669mm (38 x 38 DPI)

Impact of Human Papillomavirus age-related prevalence and vaccination levels on interpretation of cervical screening modalities – a modelling study: Supplemental material

Author: David Robert Grimes[1,2], PhD

Addresses and affiliations:

1. School of Physical Sciences, Dublin City University, Glasnevin, Dublin, Ireland (davidrobert.grimes@dcu.ie)
2. Discipline of radiation therapy, Trinity College Dublin, Trinity Centre for Health Sciences, St. James's Hospital, Dublin 8, James's Street (davidrobert.grimes@tcd.ie)

40% Vaccination uptake simulation

Results as shown in figure 1.

For peer review only

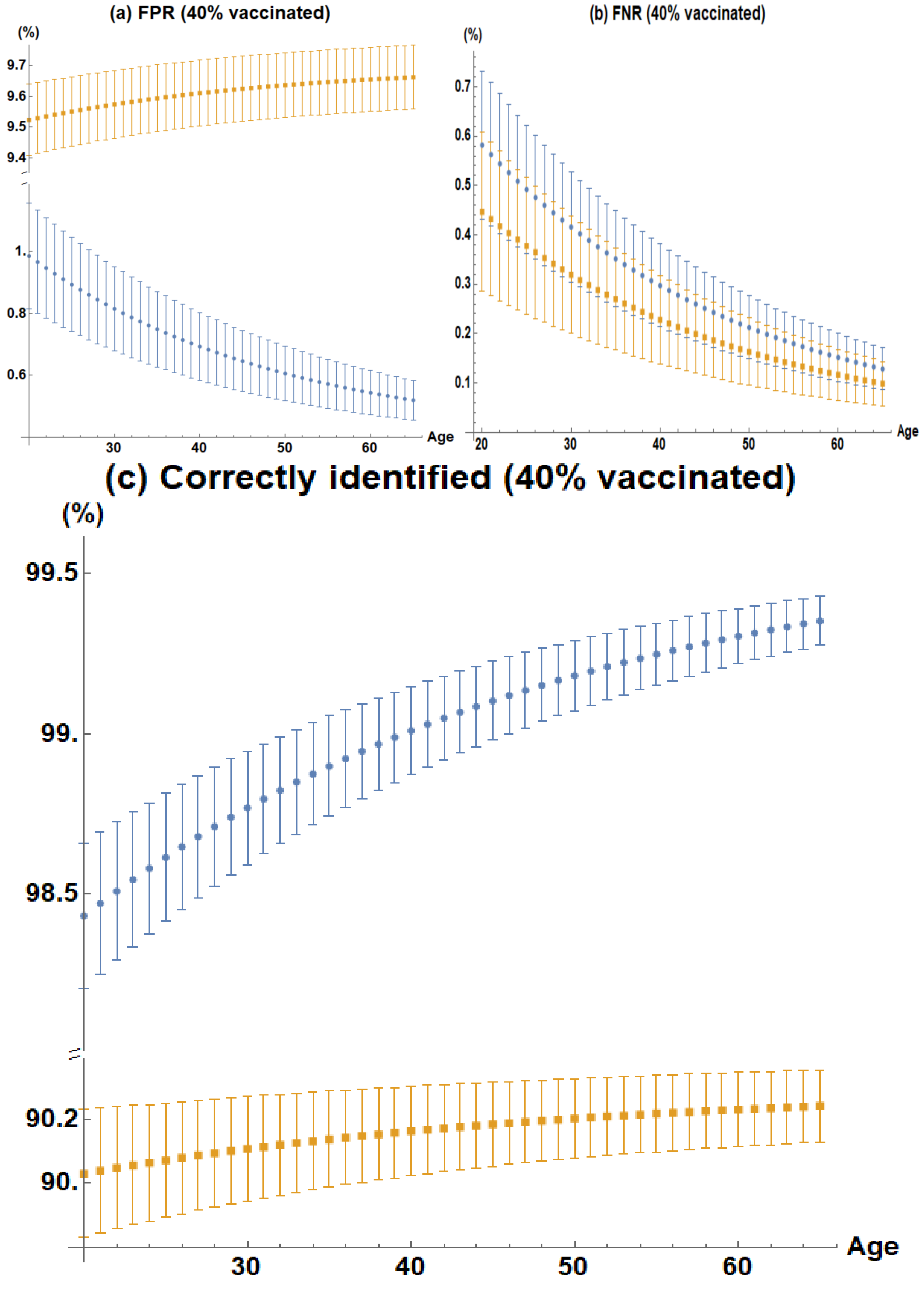

Figure 1 - 40% Vaccine uptake effect on (a) False positives (b) false negatives (c) correct identification

Age cohorts, sample size, and High risk HPV types

Study name	Number (n)	Age cohorts (N)	Hr- HPV types sampled
**Baay MF, Smits E, Tjalma WA, et al. Can cervical cancer screening be stopped at 50? The prevalence of HPV in elderly women.Int J Cancer 2004;108(2):258–61.**	1936	50-54, 55-59, 60-64, 65-69, 70+	16, 18, 31, 33, 35, 39, 45, 51, 52, 56, 58, 59, 66, 68.
**Vandenvelde C, Van Beers D. High-risk genital papillomaviruses and degree of dysplastic changes in the cervix: a prospective study by fast multiplex polymerase chain reaction in Belgium. J Med Virol 1993; 39(4):273**	323	20-30,31-40,41-50,51-60, 61-70	16,18,33
**Baay MF, Tjalma WA, Lambrechts HA, et al. Combined Pap and HPV testing in primary screening for cervical abnormalities:should HPV detection be delayed until age 35? Eur J Cancer 2005; 41(17):2704–8.**	2293	20-24, 25-29, 30-34, 35-39, 40-44, 45-50	16, 18, 31, 33, 35, 39, 45, 51, 52, 56, 58, 59, 66, 68
**Kjaer SK, van den Brule AJ, Bock JE, et al. Determinants for genital human papillomavirus (HPV) infection in 1000 randomly chosen young Danish women with normal Pap smear: are there different risk profiles for oncogenic and nononcogenic HPV types? Cancer Epidemiol Biomarkers Prev 1997;6(10):799–805**	956	20-23, 24-26, 27-29	16, 18, 31, 33, 35, 39, 45, 51, 52, 54, 56, 58, 59, 66, 68
**Boulanger JC, Sevestre H, Bauville E, et al. [Epidemiology of HPV infection]. Gynecol Obstet Fertil 2004;32(3):218–23**	3832	20-24, 25-29, 30-34, 35-39, 40-44,45-49, 50-54,55-59,60-64	16, 18, 31, 33, 35, 39, 45, 51, 52, 56, 58, 59 , 68
**Clavel C, Bory JP, Rihet S, et al. Comparative analysis of human papillomavirus detection by hybrid capture assay and routine cytologic screening to detect high-grade cervical lesions. Int J Cancer 1998; 75(4):525–8**	1028	14-20, 20-29, 30-39, 40-49, 50-59, 60-68	16, 18, 31, 33, 35, 45, 51, 52, 56
**Levert M, Clavel C, Graesslin O, et al. Human papillomavirus typing in routine cervical smears. Results from a series of 3778 patients]. Gynecol Obstet Fertil 2000;28(10):722–8**	3778	15-20, 21-30, 31-40, 41-50, 51-60	16, 18, 31, 33, 35, 39, 45, 51, 52, 56, 58, 59, 68
**Clavel C, Masure M, Bory JP, et al. Human papillomavirus testing in primary screening for the detection of high-grade cervical lesions: a study of 7932 women. Br J Cancer 2001;84(12):1616–23.**	7932	15-20, 21-30, 31-40, 41-50, 51-60	16, 18, 31, 33, 35, 39, 45, 51, 52, 56, 58, 59, 68
**Ronco G, Ghisetti V, Segnan N, et al. Prevalence of human papilloma- virus infection in women in Turin, Italy. Eur J Cancer 2005;41(2): 297–305.**	1013	25-29, 30-34, 35-39, 40-44, 45-49, 50-54, 55-59, 60-64	16, 18, 26, 31, 33, 34, 35, 39, 40, 42, 43, 44, 45, 51–59, 61, 66, 68, 70, 71, 72, 73, 81, 82, 83, 84, CP6108
**Jacobs MV, Walboomers JM, Snijders PJ, et al. Distribution of 37 mucosotropic HPV types in women with cytologically normal cervical**	3305	15-24, 25-29, 30-34, 35-39, 40-44, 45-49, 50-54, 55-	16, 18, 31, 33, 35, 39, 45, 51, 52, 56, 58, 59, 66, 68

For peer review only

**smears: the age-related patterns for high-risk and low-risk types. Int J Cancer 2000;87(2):221–7.**		59, 60-69	
**Cuzick J, Szarewski A, Terry G, et al. Human papillomavirus testing in primary cervical screening. Lancet 1995;345(8964):1533–6.**	2007	15-25, 25-29, 30-34, 35-39, 40-44, 45-49, 50-54, 55-69	16, 18, 31, 33
**Peto J, Gilham C, Deacon J, et al. Cervical HPV infection and neoplasia in a large population-based prospective study: the Manchester cohort. Br J Cancer 2004;91(5):942–53**	6128	15-19, 20-24, 25-29, 30-34, 35-39, 40-44, 45-49, 50-54, 55-69	16, 18, 26, 31, 33, 35, 39, 40, 45, 51, 52, 53, 54, 55, 56, 57, 58, 59, 66, 68, 73, 82, 83 84

Mathematical justifications for model assumptions

We can show that CIN2+ prevalence will be directly proportional to HPV prevalence, even accounting for strain types and their respective likelihood of causing lesions. Consider n strains of HPV, each constituting a proportion p of the total HPV burden. The HPV burden may be written as

$$H = p_1 + p_2 \ldots + p_n = \sum_1^n p_n.$$

We may write p as a vector, and defining u as a vector of length n with every entry equal to unity, we note through the properties of the dot product operator that it follows that

$$H = p \cdot u = \sqrt{n}|p| \cos \emptyset \rightarrow |p| = \frac{H}{\sqrt{n}\cos \emptyset}$$

where $\emptyset$ is the angle between the vectors. We can further state that each strain of HPV has a probability of becoming a CIN2+ lesion of $c_i$respectively per strain, ranging from 0 to 1. Thus, the total lesion burden is given by

$$C = c_1 p_1 + c_2 p_2 \ldots + c_n p_n = \sum_1^n c_n p_n.$$

The values for p and c can be vectors and from the properties of the dot product, we may write

$$C = p \cdot c = |p||c| \cos \theta = \frac{H\,|c| \cos \theta}{\sqrt{n}\cos \emptyset}.$$

where θ is the angle between the prevalence and risk vectors. As can be seen here, it follows that no matter the configuration of these vectors, $C \propto H$ and so the assumption is justified, regardless of the individual strains involved. The cited figure for HPV prevalence after vaccination in this work is empirical, derived from previous modelling studies, functioning simply as a multiplier to gauge the performance of HPV-reflex screening under reduced incidence of infection, independent of strain. We can however use a similar argument to the previous response to show here why the approximately linear correspondence holds. Let v be the vector efficacy of vaccination for each strain, with each entry given by

$$v_i = (1 - e_i)$$

For peer review only

where e_i is the fractional respective efficacy of the vaccine against each strain, bounded between 0 and unity. We denote an element-by-element Hadamard product, corresponding to the reduced prevalence of each HPV subtype after vaccination as $p_v = p \odot v$, and like before, the modified HPV prevalence after vaccination is given by $H_v = p_v \cdot u$. But as the Hadamard product is commutative, we can instead write this as $H_v = p \cdot (u \odot v)$. Letting $u \odot v = u_v$ for brevity, then

$$H_v = p \cdot u_v = \sqrt{u_v}|p| \cos \emptyset_{uv} \rightarrow |p| = \frac{H_v}{\sqrt{u_v} \cos \emptyset_{uv}}.$$

This can be set equal to the value for $|p|$ previously derived and rearranged to establish the identity

$$H_v = H\left(\frac{\sqrt{u_v} \cos \emptyset_{uv}}{\sqrt{n} \cos \emptyset}\right).$$

The bracketed term is effectively a constant, corresponding to the reduction in net HPV infection. This is akin to the $(1 - V)$ term in the work itself, demonstrating the empirical use of a linear reduction factor derived from previous studies is justified to ascertain the performance of screening modalities under different levels of coverage.

Model performance under inflated $\bar{h}$

In this work, the population prevalence of HPV is experimentally derived, yielding $\bar{h} = 9.4 \pm 2.3$ %, in good agreement with literature estimate of 8.4%, and with population wide European estimates of 9.8% (95% Confidence Interval: 9.2%-10.0%) and more recent results from the ATHENA study. Estimates in literature however can be someway complicated by how sampling conducted. In this model, $\bar{h}$ is data derived, but we can also force a higher value into the simulation to examine consistency ever with much higher prevalence to ascertain how screening modalities would perform with $\bar{h} = 15\%$ to investigate this. The table below shows correct, false positive, and false negative results for different age groups under both the data derived and forced HPV prevalence scenarios. As can be seen from the table, test performance results are broadly similar under both data derived and forced scenarios.

Screening accuracy per 1000 women

	$\bar{h} = 9.4 \mp 2.3\%$ (Data derived)			$\bar{h} = 15\%$ (Forced)		
**Age (modality)**	Correct	False positives	False Negatives	Correct	False positives	False Negatives
**25 (LBC)**	898 ∓ 3	94 ∓ 1	8 ∓ 3	900 ∓ 2	95 ∓ 1	5 ∓ 1
**25 (HPV-reflex)**	975 ∓ 4	15 ∓ 3	11 ∓ 3	978 ∓ 3	16 ∓ 3	7 ∓ 1
**35 (LBC)**	900 ∓ 2	95 ∓ 1	6 ∓ 2	901 ∓ 1	96 ∓ 1	4 ∓ 1
**35 (HPV-reflex)**	981 ∓ 3	12 ∓ 2	8 ∓ 2	983 ∓ 2	12 ∓ 2	5 ∓ 1
**45 (LBC)**	901 ∓ 2	95 ∓ 1	4 ∓ 2	901 ∓ 1	96 ∓ 1	3 ∓ 1
**45 (HPV-reflex)**	985 ∓ 2	9 ∓ 1	5 ∓ 2	989 ∓ 2	10 ∓ 2	3 ∓ 1
**55 (LBC)**	901 ∓ 2	96 ∓ 1	3 ∓ 1	902 ∓ 1	96 ∓ 1	2 ∓ 1
**55 (HPV-reflex)**	988 ∓ 3	8 ∓ 2	4 ∓ 1	989 ∓ 2	8 ∓ 2	2 ∓ 1

TRIPOD Checklist: Prediction Model Development

Section/Topic	Item	Checklist Item	Page
**Title and abstract**			
Title	1	Identify the study as developing and/or validating a multivariable prediction model, the target population, and the outcome to be predicted.	2
Abstract	2	Provide a summary of objectives, study design, setting, participants, sample size, predictors, outcome, statistical analysis, results, and conclusions.	2-5
**Introduction**			
Background and objectives	3a	Explain the medical context (including whether diagnostic or prognostic) and rationale for developing or validating the multivariable prediction model, including references to existing models.	2-3
	3b	Specify the objectives, including whether the study describes the development or validation of the model or both.	2-3
**Methods**			
Source of data	4a	Describe the study design or source of data (e.g., randomized trial, cohort, or registry data), separately for the development and validation data sets, if applicable.	3-4
	4b	Specify the key study dates, including start of accrual; end of accrual; and, if applicable, end of follow-up.	N/A
Participants	5a	Specify key elements of the study setting (e.g., primary care, secondary care, general population) including number and location of centres.	3-4
	5b	Describe eligibility criteria for participants.	3-4
	5c	Give details of treatments received, if relevant.	N/A
Outcome	6a	Clearly define the outcome that is predicted by the prediction model, including how and when assessed.	4-5
	6b	Report any actions to blind assessment of the outcome to be predicted.	N/A
Predictors	7a	Clearly define all predictors used in developing or validating the multivariable prediction model, including how and when they were measured.	3-5
	7b	Report any actions to blind assessment of predictors for the outcome and other predictors.	NA
Sample size	8	Explain how the study size was arrived at.	NA
Missing data	9	Describe how missing data were handled (e.g., complete-case analysis, single imputation, multiple imputation) with details of any imputation method.	NA
Statistical analysis methods	10a	Describe how predictors were handled in the analyses.	3-5
	10b	Specify type of model, all model-building procedures (including any predictor selection), and method for internal validation.	2-3
	10d	Specify all measures used to assess model performance and, if relevant, to compare multiple models.	3-7
Risk groups	11	Provide details on how risk groups were created, if done.	2-3
**Results**			
Participants	13a	Describe the flow of participants through the study, including the number of participants with and without the outcome and, if applicable, a summary of the follow-up time. A diagram may be helpful.	NA
	13b	Describe the characteristics of the participants (basic demographics, clinical features, available predictors), including the number of participants with missing data for predictors and outcome.	2-3
Model development	14a	Specify the number of participants and outcome events in each analysis.	NA
	14b	If done, report the unadjusted association between each candidate predictor and outcome.	NA
Model specification	15a	Present the full prediction model to allow predictions for individuals (i.e., all regression coefficients, and model intercept or baseline survival at a given time point).	5
	15b	Explain how to the use the prediction model.	3-5
Model performance	16	Report performance measures (with CIs) for the prediction model.	5
**Discussion**			
Limitations	18	Discuss any limitations of the study (such as nonrepresentative sample, few events per predictor, missing data).	6-7
Interpretation	19b	Give an overall interpretation of the results, considering objectives, limitations, and results from similar studies, and other relevant evidence.	6-7
Implications	20	Discuss the potential clinical use of the model and implications for future research.	6-7
**Other information**			
Supplementary information	21	Provide information about the availability of supplementary resources, such as study protocol, Web calculator, and data sets.	6-7
Funding	22	Give the source of funding and the role of the funders for the present study.	7

We recommend using the TRIPOD Checklist in conjunction with the TRIPOD Explanation and Elaboration document.

BMJ Open

Impact of Human Papillomavirus age-related prevalence and vaccination levels on interpretation of cervical screening modalities – a modelling study

Journal:	*BMJ Open*
Manuscript ID	bmjopen-2023-078551.R2
Article Type:	Original research
Date Submitted by the Author:	19-Jan-2024
Complete List of Authors:	Grimes, David; University of Oxford, & Queens University Belfast
**Primary Subject Heading**:	Public health
Secondary Subject Heading:	Sexual health, Public health
Keywords:	Mass Screening, PUBLIC HEALTH, Public health < INFECTIOUS DISEASES, Sexually Transmitted Disease

SCHOLARONE™ Manuscripts

I, the Submitting Author has the right to grant and does grant on behalf of all authors of the Work (as defined in the below author licence), an exclusive licence and/or a non-exclusive licence for contributions from authors who are: i) UK Crown employees; ii) where BMJ has agreed a CC-BY licence shall apply, and/or iii) in accordance with the terms applicable for US Federal Government officers or employees acting as part of their official duties; on a worldwide, perpetual, irrevocable, royalty-free basis to BMJ Publishing Group Ltd ("BMJ") its licensees and where the relevant Journal is co-owned by BMJ to the co-owners of the Journal, to publish the Work in this journal and any other BMJ products and to exploit all rights, as set out in our licence.

The Submitting Author accepts and understands that any supply made under these terms is made by BMJ to the Submitting Author unless you are acting as an employee on behalf of your employer or a postgraduate student of an affiliated institution which is paying any applicable article publishing charge ("APC") for Open Access articles. Where the Submitting Author wishes to make the Work available on an Open Access basis (and intends to pay the relevant APC), the terms of reuse of such Open Access shall be governed by a Creative Commons licence – details of these licences and which Creative Commons licence will apply to this Work are set out in our licence referred to above.

Other than as permitted in any relevant BMJ Author's Self Archiving Policies, I confirm this Work has not been accepted for publication elsewhere, is not being considered for publication elsewhere and does not duplicate material already published. I confirm all authors consent to publication of this Work and authorise the granting of this licence.

For peer review only

Impact of Human Papillomavirus age-related prevalence and vaccination levels on interpretation of cervical screening modalities – a modelling study

Author: David Robert Grimes[1,2], PhD

Addresses and affiliations:

1. Discipline of radiation therapy, Trinity College Dublin, Trinity Centre for Health Sciences, St. James's Hospital, Dublin 8, James's Street (davidrobert.grimes@tcd.ie)
2. School of Physical Sciences, Dublin City University, Glasnevin, Dublin, Ireland (davidrobert.grimes@dcu.ie)

Word count: 2500

Figure count: 3

Table count: 2

For peer review only

Impact of Human Papillomavirus age-related prevalence and vaccination levels on interpretation of cervical screening modalities – a modelling study

David Robert Grimes, PhD –davidrobert.grimes@tcd.ie

Abstract

Objective: Cervical screening is a life-saving intervention which reduces the incidence of and mortality from cervical cancer in the population. Human Papillomavirus (HPV) based screening modalities hold unique promise in improving screening accuracy. HPV prevalence varies markedly by age, as does resultant cervical intra-epithelial neoplasia (CIN), with higher rates recorded in younger women. With the advent of effective vaccination for HPV drastically reducing prevalence of both HPV and CIN, it is critical to model how the accuracy of different screening approaches varies with age-cohort and vaccination status. This work establishes a model for age-specific prevalence of HPV factoring in vaccine coverage, and predicts how the accuracy of common screening modalities is affected by age profile and vaccine uptake.

Design: Modelling study of HPV infection rates by age, ascertained from European cohorts prior to the introduction of vaccination. Reductions in HPV due to vaccination were estimated from the bounds predicted from multiple modelling studies, yielding a model for age-varying HPV and CIN grades 2 and above (CIN2+) prevalence.

Setting: Performance of both conventional Liquid-base cytology (LBC) screening and HPV screening with LBC reflex (HPV-reflex) were estimated under different simulated age cohorts and vaccination levels.

Participants: Simulated populations of varying age and vaccination status

Results: HPV-reflex modalities consistently result in much lower incidence of false positives than LBC testing, with an accuracy that improves even as HPV and CIN2+ rates decline.

Conclusions: HPV-reflex tests outperform LBC tests across all age profiles, resulting in greater test accuracy. This improvement is especially pronounced as HPV infection rates fall and suggests HPV-reflex modalities are robust to future changes in the epidemiology of HPV.

Strengths and weaknesses

- Extends modelling approaches to screening outcomes by incorporating the projected HPV prevalence and vaccine uptake levels of age-varying cohort.
- Provides a novel methodology for ascertaining how screening modalities will perform under likely future scenarios.
- HPV prevalence estimates are limited by assumptions efficacy and uptake of vaccination, and this can be varied with the model presented.

For peer review only

Introduction

Cervical cancer screening is a powerful life-saving intervention, associated with significant reductions in the mortality and cancer burden [1-3]. For squamous cell carcinoma in women older than 25 years, national screening paradigms have resulted in an estimated 80% reduction in mortality. Human papillomavirus (HPV) is responsible for most of these cancers, and the advent of vaccination against its oncogenic strains has already had transformative impact on both rates of HPV and cervical intra-epithelial neoplasia (CIN) [2] .

Alongside conventional Liquid based cytology (LBC) modalities for screening for CIN grades 2 and higher (CIN2+), the evolution of screening modalities to include HPV-reflex method which triage cases by HPV infection status have shown great promise [4-6]. This promise however requires careful interpretation - HPV testing is often deemed to have higher sensitivity than conventional LBC approaches, but there are nuances which demand careful consideration. The sensitivity of HPV tests is not a direct proxy to LBC sensitivity, as they measure different things. While LBC tests directly measure the presence of CIN2+, HPV tests themselves only detect the presence of high-risk strains of HPV. In younger women especially, there is high transient incidence of HPV, and using HPV infection alone as a screening modality would result in an unacceptably high false detection rate of CIN2+. False positive results not only induce anxiety [7,8] in patients but also lead to potential overtreatment, with the potential to overwhelm colposcopy services unless these are robustly designed [3,9].

Accordingly, HPV triage approaches are typically implemented, where patients testing positive for high-risk HPV receive a reflex LBC, while those testing negative do not. A recent analysis [3] demonstrated that this reflex approach has in fact a slightly lower effective sensitivity for CIN2+ (0.68, 95% Confidence interval: 0.60-0.75) than LBC testing alone (0.76, 95% Confidence interval: 0.67-0.83) in a typical population, though this seeming disadvantage is typically mitigated by expedited retesting of HPV positive results before the next screening cycle. The chief advantage of HPV-reflex tests (or LBC tests with HPV reflex) is that they hugely decrease the false positive rate, with a reflex test yielding 9.6 false positives per 1000 women (95% Confidence interval: 9.4-9.8) compared to LBC testing with 95.1 false positives per 1000 women (95% Confidence interval: 93.7- 97.0), decreasing false positive rates by approximately ten-fold and reducing overtreatment. Simplified clinical patient flow for both LBC and HPV reflex modalities are illustrated in figure 1.

Yet to date, this work has been largely considered average HPV infection rates and CIN2+ prevalence averaged across entire populations. HPV infection rates however differ by both age cohort [10] and vaccination status [11]. Younger women tend to have higher rates of both HPV infection, and CIN2+. This in turn has implications for current and future screening interpretations and planning with differing cohorts. CIN2+ prevalence is directly proportional to HPV infection [12] and will change with as infection rates change either due to aging cohorts or increased vaccine uptake. A simple dynamic model to account for this would allow accurate determination of screening performance under realistic scenarios and facilitate the planning of future screening modalities. This work accordingly establishes a model to account HPV infection rates, and CIN2+ prevalence with age cohorts and vaccine uptake, ascertaining test accuracy under all possible future scenarios.

Figure 1 - Clinical patient flow for (a) LBC screening and (b) HPV reflex screening. HPV positive, LBC negative results are frequently selected for an expedited retest outside of the normal screening interval

Methods

Modelling varying prevalence of HPV and CIN2+

For peer review only

HPV infection rates vary markedly by country and cohort, and even detection methodology. The evidence to date strongly suggests that HPV infection peaks before 30, though slightly different trends emerge in various data sets. For the purposes of this investigation, analysis was restricted to sets prior to widespread coverage of the HPV vaccine. On current estimates from USA and European data, we expect a global average of approximately 8.4% of the total population to have active HPV-infection at the time of screening [3]. This figure pertains to the total prevalence across the population, and the caveats are elucidated in the discussion.

For this work, it was crucial to further stratify this figure by age cohort. To derive an age-specific HPV prevalence rate, we considered the 2008 review by Smith et al [10] of the literature on HPV infection rates throughout the world. This review included various data sources, including these stemming from sexual infection clinics or specific high-risk populations. As these results are likely to produce high (or in some cases low) outliers which can skew analysis, we confined ourselves only to the subset of studies solely from general European screening programmes. Furthermore, this analysis only considered studies with some degree of age stratification. This resulted in 12 applicable studies [13-24] . These studies were undertaken in various cities throughout Belgium, Denmark, France, Italy, The Netherlands, and the United Kingdom between 1992 and 2005, with samples ranging from 323 women to 7932 women (mean: 2878 women). Data from these studies was then taken and weighted according to the number of study participants and smoothed, so that an age-varying phenomenological model describing HPV prevalence at age in years *a* could be ascertained with 95% confidence intervals, denoted $h_u(a)$. Details of these studies, participants, settings, and HPV subtypes tested for are given in the supplementary material.

Crucially, these estimates stem from before the advent of HPV vaccination, and accordingly map to a scenario of 0% vaccine coverage. This is a useful baseline upon which we can model the impact of vaccination on test performance. The impact of HPV vaccination on prevalence rates was derived from 29 previously published modelling studies [8], yielding an estimate for *V*, the reduction factor for HPV prevalence from given level of vaccination, as previously published [3,11]. The time-varying HPV prevalence for a given level of vaccination is thus $h(a) = (1 - V)h_u(a)$, with the mathematical justification for this simplification given in the supporting mathematical appendix. For brevity, results in this work consider the impact of vaccine rates of 0% (completely unvaccinated cohorts) and 80% (highly vaccinated cohorts) with mid-level vaccination rates of 40% given in the supplementary material. The mean value for $h(a)$ across the entire span of screening ages is given by

$$\overline{\mathrm{h}} = \frac{1}{\mathrm{a_e} - \mathrm{a_s}} \int_{a_s}^{\mathrm{a_e}} \mathrm{h}(a) \mathrm{da}\,.$$

Taking the upper bound of screening ages to be $\mathrm{a_e} = 65$ years and the lower bound to be $\mathrm{a_s} = 20$ years, the mean HPV prevalence in the cohort can be ascertained from the form of the phenomenological function. The CIN2/3 prevalence is proportional to the rates of HPV infection, with a justifying argument for this given in the mathematical supplementary material. It follows then that a simple scaling function should be predictive of prevalence rates for CIN2+. If the known population average of CIN2+ is $\overline{\mathrm{p}}$, then age / vaccine varying CIN2+ prevalence is accordingly given by

$$p(a) = \left(\frac{\overline{p}}{\overline{h}}\right) h(a).$$

Interpretation of screening results with varying HPV prevalence and vaccine uptake.

For peer review only

With age and vaccination specific CIN2/3 prevalence known, one can explicitly calculate false positive rates, false negative rates, and over-all correct identifications using previously published methods. For Liquid based cytology (LBC), the false positive (fp_l), false negative (fn_l), and over-all correct identification rates (t_l) are related to CIN2/3 prevalence and the sensitivity (s_{nl}) and specificity (s_{pl}) of LBC testing, given respectively by the relationships

$$fp_l(a) = (1 - p(a))(1 - s_{pl})$$

$$fn_l(a) = p(a)(1 - s_{nl})$$

$$t_l(a) = 1 - (f_{pl}(a) + f_{nl}(a)).$$

For HPV reflex testing, false positive rates (fp_r), false negative rates (fn_r),, and over-all correct identification rate (t_r) depend additionally on HPV prevalence, the sensitivity of HPV testing (s_{nh}), the specificity of HPV testing (s_{ph}) and the fraction of CIN2+ cases associated with detectable HPV, *v*. These equations are derived from those previously published[3], and are respectively

$$fp_r(a) = (1 - s_{pl})(s_{nh}(h(a) - p(a)v) + (1 - s_{ph})(1 - h(a) - p(a)(1 - v)))$$

$$fn_r(a) = vp(a)(1 - s_{nl}s_{nh}) + p(a)(1 - v)(1 - s_{nl}(1 - s_{ph}))$$

$$t_r(a) = 1 - (f_{pl}(a) + f_{nl}(a)).$$

Literature values used for all modelling simulations are given in table 1.

Table 1: Simulation values and source

Parameter	Values (95% CI where available)
Average prevalence of CIN2/3 in full population ($\bar{p}$)	0.02[6]
Sensitivity of LBC test to CIN2+ (s_{nl})	0.76 ± 0.04 [6]
Specificity of LBC test to CIN2+ (s_{pl})	0.903 ± 0.001 [6]
Sensitivity of HPV test to High-Risk HPV (s_{nh})	$0.947\ (-)$ [3]
Specificity of HPV test to High-Risk HPV (s_{ph})	0.96 ± 0.001 [6]
CIN2/3 attributable to testable hr-HPV (ν)	$0.95(-)$ [3]
Reduction in HPV at 40% vaccine coverage (V_{40})	0.53 ± 0.06 [3,8]
Reduction in HPV at 80% vaccine coverage (V_{80})	0.93 ± 0.026 [3,8]

For peer review only

Patient and Public Involvement

None. It was not appropriate or possible to involve patients or the public in the design, or conduct, or reporting, or dissemination plans of this research.

Results

Modelling varying prevalence of HPV and CIN2+

Age-related HPV prevalence from 12 included studies were found to approximately obey an exponential decay type-model from ages 20-65 of the form $A\exp(-\beta t)$ as illustrated in figure 1 ($A = 35.81 \pm 4.91$, $\beta = 0.0336 \pm 0.0045\ yr^{-1}$, 95% confidence interval). This envelope with weighed study data points are shown in figure 1. The estimate for mean HPV infection rate in unvaccinated populations across all age ranges was $\bar{h} = 9.4 \pm 2.3\,\%$, in good agreement with literature estimate of 8.4%, and with population wide European estimates of 9.8% [25] (95% Confidence Interval: 9.2%-10.0%) and from the ATHENA study [26] (10.5%, 95% Confidence Interval: 10.3%-10.7%) . CIN2+ rates by age cohort from inferred from this as per the model outline.

Figure 2 - Weighted fits from 12 European population studies with exponential fit and confidence intervals.

Interpretation of screening results with varying HPV prevalence and vaccine uptake

Figure 2 depicts how the accuracy of LBC and HPV-reflex modalities (in terms of true results, false positives, and false negatives) change with age in both unvaccinated and 80% vaccinated populations. HPV reflex modalities consistently outperform LBC modalities for all age cohorts and vaccine uptake levels. Table 2 depicts a selection of results for varying age profiles and vaccine uptake, with additional Simulation results for 40% vaccine coverage are given in the supplementary material.

Figure 3: Performance of HPV-reflex and LBC screening modalities with age. Percentage correct results shown for (a) unvaccinated populations and (b) 80% vaccine uptake populations. False positive rates of both modalities with age shown for (c) unvaccinated populations and (d) 80% vaccine uptake populations. False negative rates depicted for (e) unvaccinated populations and (f) 80% vaccinated populations. Note the broken Y-axis in (a)-(d) inclusive

Table 2: Modelled test performance at specific ages and vaccination levels

	LBC			HPV reflex		
**Age profile**	***False Negative %***	***False Positive %***	***Correct Result %***	***False Negative %***	***False Positive %***	***Correct Result %***
Age 25						
**0% Vaccinated**	0.8 ± 0.28	9.38 ± 0.14	89.1 ± 0.31	1.05 ± 0.26	1.46 ± 0.27	97.50 ± 0.40
**40% Vaccinated**	0.38 ± 0.14	9.55 ± 0.11	90.07 ± 0.18	0.49 ± 0.13	1.61 ± 0.26	97.89 ± 0.29
**80% Vaccinated**	0.06 ± 0.03	9.68 ± 0.10	90.27 ± 0.11	0.07 ± 0.03	1.73 ± 0.26	98.20 ± 0.26
Age 35						
**0% Vaccinated**	0.57 ± 0.21	9.47 ± 0.12	89.95 ± 0.24	0.75 ± 0.20	1.16 ± 0.23	98.10 ± 0.30

For peer review only

**40% Vaccinated**	0.27 ± 0.10	9.59 ± 0.11	90.14 ± 0.15	0.35 ± 0.10	1.26 ± 0.22	98.38 ± 0.24
**80% Vaccinated**	0.04 ± 0.02	9.68 ± 0.10	90.28 ± 0.10	0.05 ± 0.02	1.35 ± 0.22	98.60 ± 0.22
Age 45						
**0% Vaccinated**	0.41 ± 0.16	9.54 ± 0.12	90.05 ± 0.20	0.53 ± 0.15	0.94 ± 0.19	98.53 ± 0.24
**40% Vaccinated**	0.19 ± 0.08	9.62 ± 0.11	90.18 ± 0.11	0.25 ± 0.07	1.01 ± 0.18	98.73 ± 0.20
**80% Vaccinated**	0.03 ± 0.02	9.69 ± 0.10	90.28 ± 0.10	0.04 ± 0.01	1.07 ± 0.18	98.89 ± 0.18
Age 55						
**0% Vaccinated**	0.29 ± 0.12	9.58 ± 0.11	90.12 ± 0.16	0.38 ± 0.11	0.78 ± 0.16	98.84 ± 0.19
**40% Vaccinated**	0.14 ± 0.06	9.65 ± 0.10	90.22 ± 0.12	0.18 ± 0.06	0.84 ± 0.15	98.99 ± 0.16
**80% Vaccinated**	0.02 ± 0.01	9.69 ± 0.10	90.29 ± 0.10	0.03 ± 0.01	0.88 ± 0.15	99.10 ± 0.10

Discussion

In all simulated cases with realistic parameters, HPV-reflex testing correctly identifies a higher proportion of cases ($97.1 \pm 0.4\%$ to $99.6 \pm 0.2\%$) relative to LBC testing for the same scenarios ($89.7 \pm 0.4\%$ to $90.3 \pm 0.1\%$). This is seen clearly in Figure 3(a) and (b), and detailed in table 2. The superior performance of HPV-reflex manifests chiefly due to markedly lower false positive rates. In all scenarios, LBC false positive rates exceed HPV-reflex values for all scenarios by a factor of 5.6 to 23.8. This analysis focused on a comparison of HPV-reflex to LBC testing. It did not explicitly consider co-testing, where both LBC and HPV screening are performed and a positive on either leads to colposcopy, as this modality leads to unacceptably high false positives results, although it is possible in principle to extend the analysis here to consider how this scenario would vary by age cohort and vaccination status.

This difference is particularly pronounced in vaccinated cohorts, as false positives decline markedly with lower HPV prevalence in HPV-reflex testing. By contrast, false positive rates slightly increase in vaccinated cohorts with LBC testing, as seen in figure 3(c)-(d). This Is expected, as the limit of LBC false positive rate as CIN2+ prevalence approaches zero is simply $1 - s_{pl}$, yielding a theoretical maximum possible false positive rate for LBC of approximately 9.7%. By contrast, false positives decline with reflex with HPV, resulting in a theoretical minimum false positive rate of $(1 - s_{pl})(1 - s_{ph})$, or a minimum value of approximately 0.39%.

For both LBC and reflex testing, false negatives decline with decreasing HPV prevalence. LBC testing does have a slightly lower false negative rate than HPV-reflex testing, but this is marginal and overlaps with the performance envelope for HPV-reflex testing. In principle, LBC testing is more sensitive to instances of CIN2+ than reflex screening, but it results in far more false positives and unnecessary referrals to colposcopy. As HPV detection sensitivity is so high, expedited retesting can be employed when a patient tests positive for a high-risk strain of HPV, and negative on LBC. This is likely to more than compensate for the marginally higher false negative rate of reflex testing, while preserving the crucial benefit and over-all accuracy. Expedited retesting is already implemented for HPV positive results in several screening programmes, including the Irish National screening programme CervicalCheck [27] . where HPV positive but cytology negative patients are recalled for repeat screening after one year rather than three or five. Such approaches maintain the benefits of HPV-reflex without the potential drawback of lower net-sensitivity.

One limitation of the current study was the assumption that CIN2+ prevalence corresponds linearly to HPV prevalence, yielding an approximately constant proportion with age cohort. This is a reasonable

For peer review only

assumption as justified in the mathematical appendix, but there are several important caveats that need to be considered. Firstly, the proportion estimated in this work pivots on how well the populations in the HPV and CIN2+ datasets correspond to one another. While these data sets were chosen to avoid the potentially skewing influence of elective sexually transmitted disease clinic settings, there may be other confounding reasons why they are not representative, in which case estimates of $p(a)$ might be biased.

In addition, the proportionality itself might vary with time for biological reasons. While younger women for example might have a higher rate of HPV, it is conceivable that they have better mechanisms of clearance, and so proportionally might have a lower fraction of CIN2+ compared to older cohorts. In the absence of clear data, this is of course speculative but the existence of any such age-specific variation in clearance rate would potentially impact the estimates in this work.

It is worth briefly discussing the mean HPV infection rate in unvaccinated populations across all age ranges, calculated as $\bar{h} = 9.4 \pm 2.3\ \%$ in this work. This figure is in good agreement with literature estimate of 8.4% as described, and with population wide European estimates of 9.8% [25] (95% Confidence Interval: 9.2%-10.0%) and more recent results from the ATHENA study [26]. Estimates in literature however can be someway complicated by how sampling conducted – samples drawn from opportunistic testing or from specific environments like sexual health clinics, or samples skewed heavily towards youth may tend to over-estimate prevalence. Bruni et al [25], for example, in their meta-analysis reported a prevalence of 14.2% (ranging from 8.8% in Southern Europe to 21.4% in Eastern Europe.

These estimates are readily confounded by the age and selection bias of subjects in the underlying studies (for example, studies disproportionately consisting of younger women or opportunistic testing), hence the disparity with the reported population prevalence even in the same work. In this model, $\bar{h}$ is data derived, but we can also force a higher value into the simulation to examine consistency ever with much higher prevalence. The supplementary material contains a simulation assigning $\bar{h} = 15\%$ to investigate this, and demonstrates that even this much higher contrived estimate has only negligible impact on false positive and negative rates.

It is important to note that this analysis did not consider the impacts of follow-up between screening rounds, nor the effects intervals between screening rounds, yielding instead an age-specific point estimate of false positive and negative rates in isolation. The cumulative false positive and negative rate over multiple screening rounds will vary depending on the intervals between screening, the rate of HPV clearance, and the modalities employed. This is an involved question beyond the scope of this work, and the impacts of repeated screening on false negative and positive rates require dedicated future analysis to answer fully.

In consequence, HPV-reflex testing is calculated to be more robust to changing age and vaccine status than LBC testing and will increase further in accuracy as HPV infection decreases due to vaccination. This is an important consideration for the planning and interpretation of future screening and colposcopy services, which need to be robust for both age variation of HPV infection, and the eventuality where HPV infection is virtually eradicated due to increased uptake of the HPV vaccine. This is especially critical considering the World Health Organisations' cervical cancer elimination strategy 90-70-90 targets. These proposed measures [28], to be put in place across nations worldwide by 2030 to eliminate cervical cancer, comprise 90% of girls vaccinated against HPV by age 15, 70% of women screened by age 35, and 90% of people identified with cervical disease treated. The results of this analysis strongly suggest that HPV-reflex screening approaches are robust to this changing

For peer review only

epidemiology of HPV infection, and will continue to perform strongly even as HPV infections fall with increasing vaccine uptake.

Competing interests statement

Dr Grimes reported that he has written articles on both screening and HPV vaccination for popular audiences, receiving no industry funding for these articles. He also reports that he has consulted for public health bodies and charities in Europe on screening. He also reported receiving paid royalties for a popular science book and receiving fees and honoraria for contributed articles, fact-checking, public talks, and media appearances.

Ethical approval statement

This work involved no patient or personal data and only used publicly available and previously published prevalence data. Accordingly, no ethical approval was required for this work.

Funding statement

This work was funded by grant number 214461/Z/18/Z from the Wellcome Trust. The funder had no role in the design and conduct of the study; collection, management, analysis, and interpretation of the data; preparation, review, or approval of the manuscript; and decision to submit the manuscript for publication.

Data sharing statement

Source code for the simulations herein are hosted online at https://github.com/drg85/CervScreenAgeVax

Authors' contributions

DRG conceived the model, analysed the published data, performed the analysis, reported the results and wrote the manuscript.

References

1. Peto, J., Gilham, C., Fletcher, O., & Matthews, F. E. (2004). The cervical cancer epidemic that screening has prevented in the UK. The Lancet, 364(9430), 249-256.
2. Hall, M. T., Simms, K. T., Lew, J. B., Smith, M. A., Brotherton, J. M., Saville, M., ... & Canfell, K. (2019). The projected timeframe until cervical cancer elimination in Australia: a modelling study. The Lancet Public Health, 4(1), e19-e27.
3. Grimes, D. R., Corry, E. M., Malagón, T., O'Riain, C., Franco, E. L., & Brennan, D. J. (2021). Modeling cervical cancer screening strategies with varying levels of human papillomavirus vaccination. JAMA network open, 4(6), e2115321-e2115321.
4. Franco EL, Mahmud SM, Tota J, Ferenczy A, Coutlée F. The expected impact of HPV vaccination on the accuracy of cervical cancer screening: the need for a paradigm change. Arch Med Res. 2009;40(6):478-485
5. Ronco G, Dillner J, Elfström KM, et al; International HPV screening working group. Efficacy of HPV-based screening for prevention of invasive cervical cancer: follow-up of four European randomised controlled trials. Lancet. 2014;383(9916):524-532. doi:10.1016/S0140-6736(13)62218-7

For peer review only

6. Koliopoulos G, Nyaga VN, Santesso N, et al. Cytology versus HPV testing for cervical cancer screening in the general population. Cochrane Database Syst Rev. 2017;8:CD008587. doi:10.1002/14651858.CD008587.pub2
7. Wittenborn, J., Wagels, L., Kupec, T., Iborra, S., Najjari, L., & Stickeler, E. (2022). Anxiety in women referred for colposcopy: a prospective observational study. Archives of gynecology and obstetrics, 1-6.
8. Kohler, R. E., Hemler, J., Wagner, R. B., Sullivan, B., Macenat, M., Tagai, E. K., ... & Hudson, S. V. (2023). Confusion and anxiety in between abnormal cervical cancer screening results and colposcopy:"The land of the unknown". Patient Education and Counseling, 114, 107810.
9. Wright, J. D., Chen, L., Tergas, A. I., Melamed, A., Clair, C. M. S., Hou, J. Y., ... & Hershman, D. L. (2021). Overuse of cervical cancer screening tests among women with average risk in the United States from 2013 to 2014. JAMA Network Open, 4(4), e218373-e218373.
10. Smith, J. S., Melendy, A., Rana, R. K., & Pimenta, J. M. (2008). Age-specific prevalence of infection with human papillomavirus in females: a global review. Journal of Adolescent Health, 43(4), S5-e1.
11. Brisson, M., Bénard, É., Drolet, M., Bogaards, J. A., Baussano, I., Vänskä, S., ... & Walsh, C. (2016). Population-level impact, herd immunity, and elimination after human papillomavirus vaccination: a systematic review and meta-analysis of predictions from transmission-dynamic models. The Lancet Public Health, 1(1), e8-e17.
12. Ting J, Kruzikas DT, Smith JS. A global review of age-specific and overall prevalence of cervical lesions. Int J Gynecol Cancer. 2010 Oct;20(7):1244-9. doi: 10.1111/igc.0b013e3181f16c5f. PMID: 21495248.
13. Baay MF, Smits E, Tjalma WA, et al. Can cervical cancer screening be stopped at 50? The prevalence of HPV in elderly women.Int J Cancer 2004;108(2):258–61.
14. Vandenvelde C, Van Beers D. High-risk genital papillomaviruses and degree of dysplastic changes in the cervix: a prospective study by fast multiplex polymerase chain reaction in Belgium. J Med Virol 1993; 39(4):273
15. Baay MF, Tjalma WA, Lambrechts HA, et al. Combined Pap and HPV testing in primary screening for cervical abnormalities:should HPV detection be delayed until age 35? Eur J Cancer 2005; 41(17):2704–8.
16. Kjaer SK, van den Brule AJ, Bock JE, et al. Determinants for genital human papillomavirus (HPV) infection in 1000 randomly chosen young Danish women with normal Pap smear: are there different risk profiles for oncogenic and nononcogenic HPV types? Cancer Epidemiol Biomarkers Prev 1997;6(10):799–805
17. Boulanger JC, Sevestre H, Bauville E, et al. [Epidemiology of HPV infection]. Gynecol Obstet Fertil 2004;32(3):218–23
18. Clavel C, Bory JP, Rihet S, et al. Comparative analysis of human papillomavirus detection by hybrid capture assay and routine cytologic screening to detect high-grade cervical lesions. Int J Cancer 1998; 75(4):525–8
19. Levert M, Clavel C, Graesslin O, et al. Human papillomavirus typing in routine cervical smears. Results from a series of 3778 patients]. Gynecol Obstet Fertil 2000;28(10):722–8
20. Clavel C, Masure M, Bory JP, et al. Human papillomavirus testing in primary screening for the detection of high-grade cervical lesions: a study of 7932 women. Br J Cancer 2001;84(12):1616–23.
21. Ronco G, Ghisetti V, Segnan N, et al. Prevalence of human papilloma- virus infection in women in Turin, Italy. Eur J Cancer 2005;41(2): 297–305.

For peer review only

22. Jacobs MV, Walboomers JM, Snijders PJ, et al. Distribution of 37 mucosotropic HPV types in women with cytologically normal cervical smears: the age-related patterns for high-risk and low-risk types. Int J Cancer 2000;87(2):221–7.
23. Cuzick J, Szarewski A, Terry G, et al. Human papillomavirus testing in primary cervical screening. Lancet 1995;345(8964):1533–6.
24. Peto J, Gilham C, Deacon J, et al. Cervical HPV infection and neoplasia in a large population-based prospective study: the Manchester cohort. Br J Cancer 2004;91(5):942–53
25. Bruni, L., Diaz, M., Castellsagué, M., Ferrer, E., Bosch, F. X., & de Sanjosé, S. (2010). Cervical human papillomavirus prevalence in 5 continents: meta-analysis of 1 million women with normal cytological findings. Journal of Infectious Diseases, 202(12), 1789-1799.
26. Wright, T. C., Stoler, M. H., Behrens, C. M., Sharma, A., Zhang, G., & Wright, T. L. (2015). Primary cervical cancer screening with human papillomavirus: end of study results from the ATHENA study using HPV as the first-line screening test. Gynecologic oncology, 136(2), 189-197.
27. CervicalCheck National Screening Programme, Health Service Executive Ireland. Cervical Screening results and recommendations table CS/PUB/LAB-2 Rev. 12 (2021) – Available online from https://www.cervicalcheck.ie/_fileupload/Health-professionals/CervicalScreeningResultsandRecommendations.pdf
28. Wilailak, S., Kengsakul, M., & Kehoe, S. (2021). Worldwide initiatives to eliminate cervical cancer. International Journal of Gynecology & Obstetrics, 155, 102-106.

For peer review only

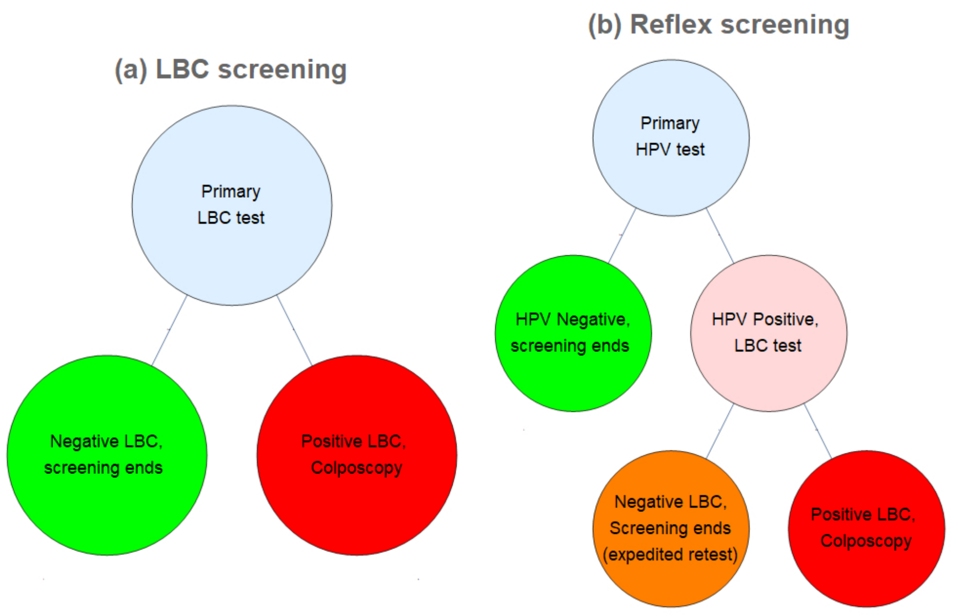

Clinical patient flow for (a) LBC screening and (b) HPV reflex screening. HPV positive, LBC negative results are frequently selected for an expedited retest outside of the normal screening interval

685x441mm (38 x 38 DPI)

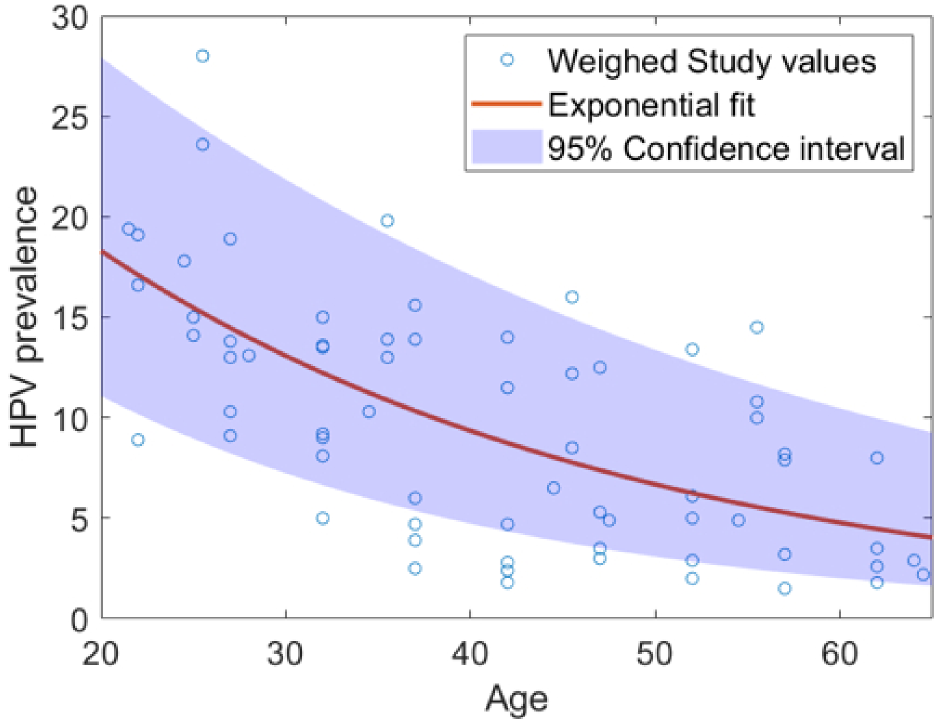

Figure 2 - Weighted fits from 12 European population studies with exponential fit and confidence intervals.

359x279mm (38 x 38 DPI)

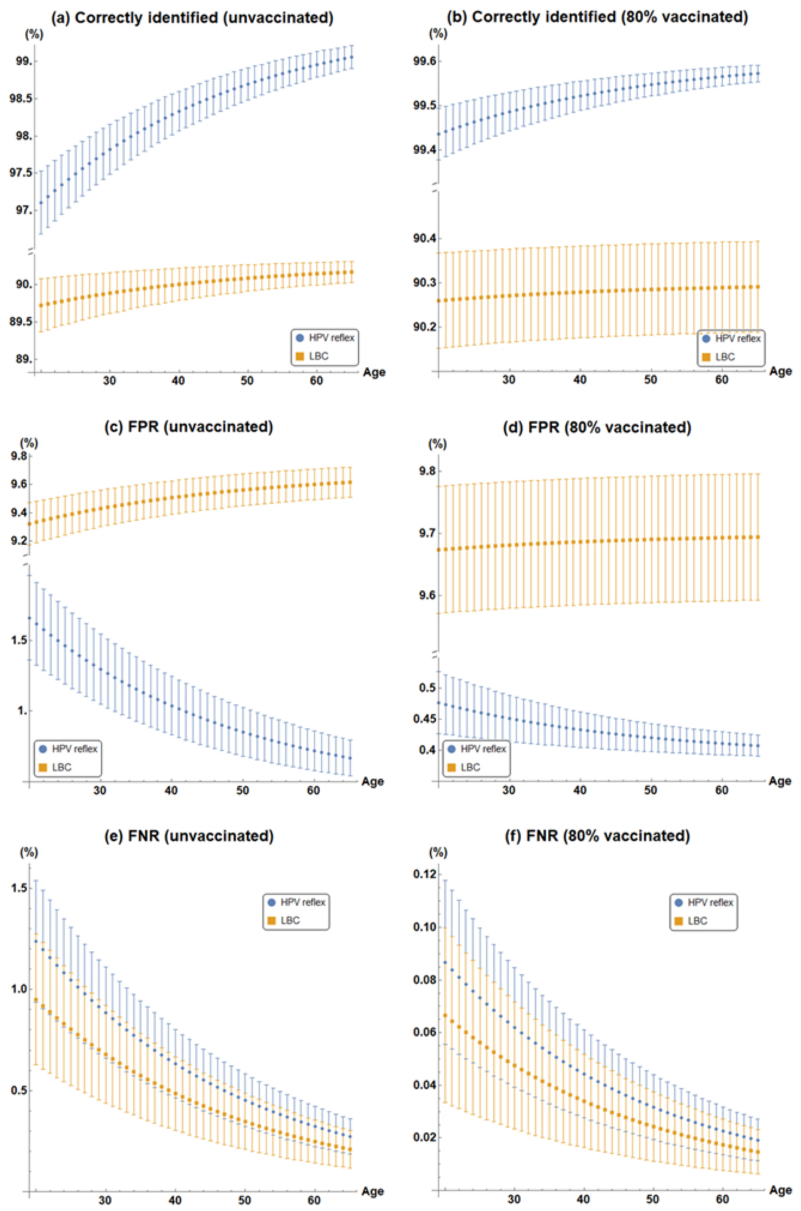

Figure 3: Performance of HPV-reflex and LBC screening modalities with age. Percentage correct results shown for (a) unvaccinated populations and (b) 80% vaccine uptake populations. False positive rates of both modalities with age shown for (c) unvaccinated populations and (d) 80% vaccine uptake populations. False negative rates depicted for (e) unvaccinated populations and (f) 80% vaccinated populations. Note the broken Y-axis in (a)-(d) inclusive

439x669mm (38 x 38 DPI)

Impact of Human Papillomavirus age-related prevalence and vaccination levels on interpretation of cervical screening modalities – a modelling study: Supplemental material

Author: David Robert Grimes[1,2], PhD

Addresses and affiliations:

1. School of Physical Sciences, Dublin City University, Glasnevin, Dublin, Ireland (**davidrobert.grimes@dcu.ie**)
2. Discipline of radiation therapy, Trinity College Dublin, Trinity Centre for Health Sciences, St. James's Hospital, Dublin 8, James's Street (**davidrobert.grimes@tcd.ie**)

40% Vaccination uptake simulation

Results as shown in figure 1.

For peer review only

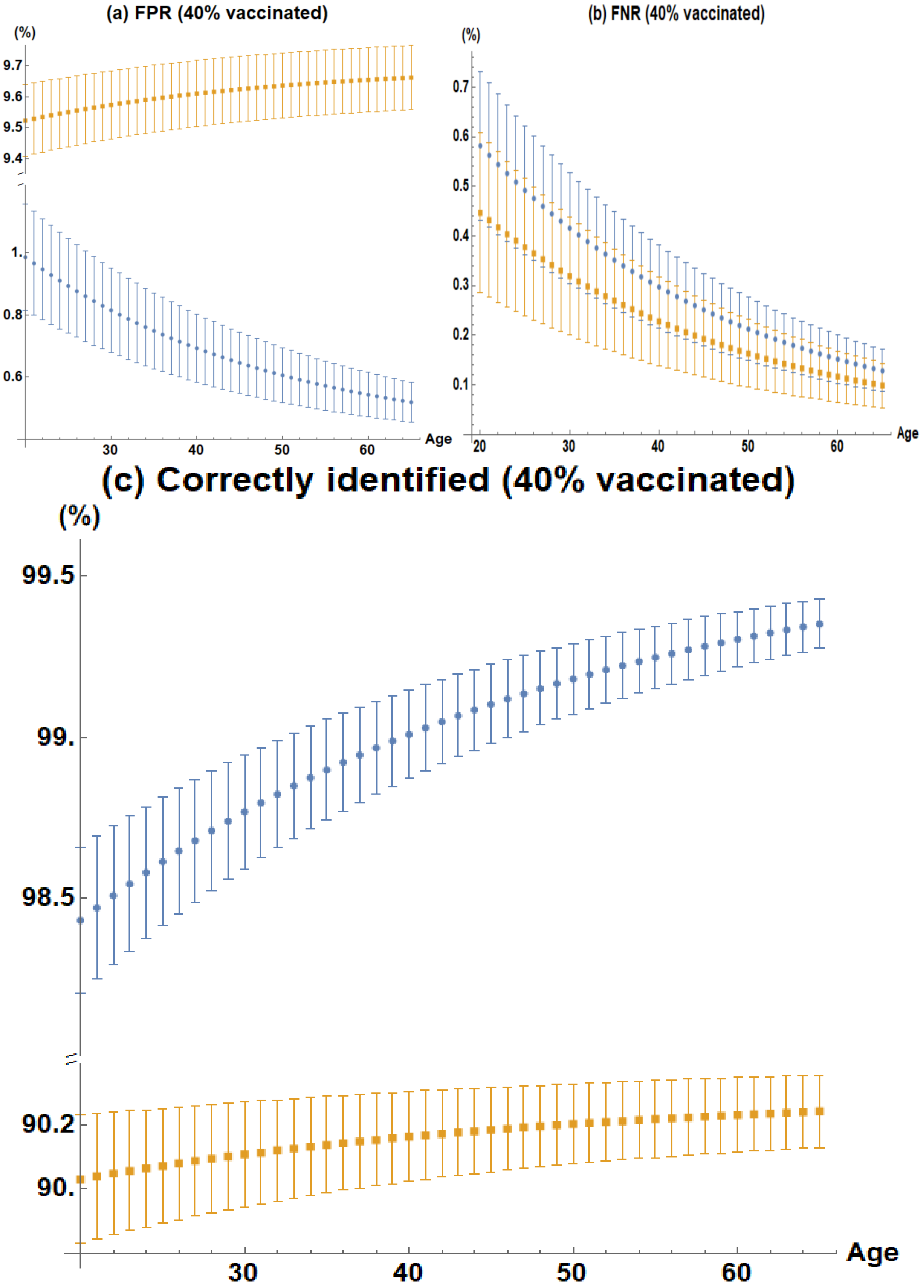

Figure 1 - 40% Vaccine uptake effect on (a) False positives (b) false negatives (c) correct identification

Age cohorts, sample size, and High risk HPV types

Study name	Number (n)	Age cohorts (N)	Hr- HPV types sampled
**Baay MF, Smits E, Tjalma WA, et al. Can cervical cancer screening be stopped at 50? The prevalence of HPV in elderly women.Int J Cancer 2004;108(2):258–61.**	1936	50-54, 55-59, 60-64, 65-69, 70+	16, 18, 31, 33, 35, 39, 45, 51, 52, 56, 58, 59, 66, 68.
**Vandenvelde C, Van Beers D. High-risk genital papillomaviruses and degree of dysplastic changes in the cervix: a prospective study by fast multiplex polymerase chain reaction in Belgium. J Med Virol 1993; 39(4):273**	323	20-30,31-40,41-50,51-60, 61-70	16,18,33
**Baay MF, Tjalma WA, Lambrechts HA, et al. Combined Pap and HPV testing in primary screening for cervical abnormalities:should HPV detection be delayed until age 35? Eur J Cancer 2005; 41(17):2704–8.**	2293	20-24, 25-29, 30-34, 35-39, 40-44, 45-50	16, 18, 31, 33, 35, 39, 45, 51, 52, 56, 58, 59, 66, 68
**Kjaer SK, van den Brule AJ, Bock JE, et al. Determinants for genital human papillomavirus (HPV) infection in 1000 randomly chosen young Danish women with normal Pap smear: are there different risk profiles for oncogenic and nononcogenic HPV types? Cancer Epidemiol Biomarkers Prev 1997;6(10):799–805**	956	20-23, 24-26, 27-29	16, 18, 31, 33, 35, 39, 45, 51, 52, 54, 56, 58, 59, 66, 68
**Boulanger JC, Sevestre H, Bauville E, et al. [Epidemiology of HPV infection]. Gynecol Obstet Fertil 2004;32(3):218–23**	3832	20-24, 25-29, 30-34, 35-39, 40-44,45-49, 50-54,55-59,60-64	16, 18, 31, 33, 35, 39, 45, 51, 52, 56, 58, 59 , 68
**Clavel C, Bory JP, Rihet S, et al. Comparative analysis of human papillomavirus detection by hybrid capture assay and routine cytologic screening to detect high-grade cervical lesions. Int J Cancer 1998; 75(4):525–8**	1028	14-20, 20-29, 30-39, 40-49, 50-59, 60-68	16, 18, 31, 33, 35, 45, 51, 52, 56
**Levert M, Clavel C, Graesslin O, et al. Human papillomavirus typing in routine cervical smears. Results from a series of 3778 patients]. Gynecol Obstet Fertil 2000;28(10):722–8**	3778	15-20, 21-30, 31-40, 41-50, 51-60	16, 18, 31, 33, 35, 39, 45, 51, 52, 56, 58, 59, 68
**Clavel C, Masure M, Bory JP, et al. Human papillomavirus testing in primary screening for the detection of high-grade cervical lesions: a study of 7932 women. Br J Cancer 2001;84(12):1616–23.**	7932	15-20, 21-30, 31-40, 41-50, 51-60	16, 18, 31, 33, 35, 39, 45, 51, 52, 56, 58, 59, 68
**Ronco G, Ghisetti V, Segnan N, et al. Prevalence of human papilloma- virus infection in women in Turin, Italy. Eur J Cancer 2005;41(2): 297–305.**	1013	25-29, 30-34, 35-39, 40-44, 45-49, 50-54, 55-59, 60-64	16, 18, 26, 31, 33, 34, 35, 39, 40, 42, 43, 44, 45, 51–59, 61, 66, 68, 70, 71, 72, 73, 81, 82, 83, 84, CP6108
**Jacobs MV, Walboomers JM, Snijders PJ, et al. Distribution of 37 mucosotropic HPV types in women with cytologically normal cervical**	3305	15-24, 25-29, 30-34, 35-39, 40-44, 45-49, 50-54, 55-	16, 18, 31, 33, 35, 39, 45, 51, 52, 56, 58, 59, 66, 68

**smears: the age-related patterns for high-risk and low-risk types. Int J Cancer 2000;87(2):221–7.**		59, 60-69	
**Cuzick J, Szarewski A, Terry G, et al. Human papillomavirus testing in primary cervical screening. Lancet 1995;345(8964):1533–6.**	2007	15-25, 25-29, 30-34, 35-39, 40-44, 45-49, 50-54, 55-69	16, 18, 31, 33
**Peto J, Gilham C, Deacon J, et al. Cervical HPV infection and neoplasia in a large population-based prospective study: the Manchester cohort. Br J Cancer 2004;91(5):942–53**	6128	15-19, 20-24, 25-29, 30-34, 35-39, 40-44, 45-49, 50-54, 55-69	16, 18, 26, 31, 33, 35, 39, 40, 45, 51, 52, 53, 54, 55, 56, 57, 58, 59, 66, 68, 73, 82, 83 84

Mathematical justifications for model assumptions

We can show that CIN2+ prevalence will be directly proportional to HPV prevalence, even accounting for strain types and their respective likelihood of causing lesions. Consider n strains of HPV, each constituting a proportion p of the total HPV burden. The HPV burden may be written as

$$H = p_1 + p_2 \dots + p_n = \sum_1^n p_n.$$

We may write p as a vector, and defining u as a vector of length n with every entry equal to unity, we note through the properties of the dot product operator that it follows that

$$H = p \cdot u = \sqrt{n}|p| \cos \emptyset \to |p| = \frac{H}{\sqrt{n} \cos \emptyset}$$

where $\emptyset$ is the angle between the vectors. We can further state that each strain of HPV has a probability of becoming a CIN2+ lesion of $c_i$respectively per strain, ranging from 0 to 1. Thus, the total lesion burden is given by

$$C = c_1 p_1 + c_2 p_2 \dots + c_n p_n = \sum_1^n c_n p_n.$$

The values for p and c can be vectors and from the properties of the dot product, we may write

$$C = p \cdot c = |p||c| \cos \theta = \frac{H\, |c| \cos \theta}{\sqrt{n} \cos \emptyset}.$$

where θ is the angle between the prevalence and risk vectors. As can be seen here, it follows that no matter the configuration of these vectors, $C \propto H$ and so the assumption is justified, regardless of the individual strains involved. The cited figure for HPV prevalence after vaccination in this work is empirical, derived from previous modelling studies, functioning simply as a multiplier to gauge the performance of HPV-reflex screening under reduced incidence of infection, independent of strain. We can however use a similar argument to the previous response to show here why the approximately linear correspondence holds. Let v be the vector efficacy of vaccination for each strain, with each entry given by

$$v_i = (1 - e_i)$$

For peer review only

where e_i is the fractional respective efficacy of the vaccine against each strain, bounded between 0 and unity. We denote an element-by-element Hadamard product, corresponding to the reduced prevalence of each HPV subtype after vaccination as $p_v = p \odot v$, and like before, the modified HPV prevalence after vaccination is given by $H_v = p_v \cdot u$. But as the Hadamard product is commutative, we can instead write this as $H_v = p \cdot (u \odot v)$. Letting $u \odot v = u_v$ for brevity, then

$$H_v = p \cdot u_v = \sqrt{u_v}|p| \cos \emptyset_{uv} \rightarrow |p| = \frac{H_v}{\sqrt{u_v} \cos \emptyset_{uv}}.$$

This can be set equal to the value for $|p|$ previously derived and rearranged to establish the identity

$$H_v = H \left(\frac{\sqrt{u_v} \cos \emptyset_{uv}}{\sqrt{n} \cos \emptyset} \right).$$

The bracketed term is effectively a constant, corresponding to the reduction in net HPV infection. This is akin to the $(1 - V)$ term in the work itself, demonstrating the empirical use of a linear reduction factor derived from previous studies is justified to ascertain the performance of screening modalities under different levels of coverage.

Model performance under inflated $\bar{h}$

In this work, the population prevalence of HPV is experimentally derived, yielding $\bar{h} = 9.4 \pm 2.3$ %, in good agreement with literature estimate of 8.4%, and with population wide European estimates of 9.8% (95% Confidence Interval: 9.2%-10.0%) and more recent results from the ATHENA study. Estimates in literature however can be someway complicated by how sampling conducted. In this model, $\bar{h}$ is data derived, but we can also force a higher value into the simulation to examine consistency ever with much higher prevalence to ascertain how screening modalities would perform with $\bar{h} = 15\%$ to investigate this. The table below shows correct, false positive, and false negative results for different age groups under both the data derived and forced HPV prevalence scenarios. As can be seen from the table, test performance results are broadly similar under both data derived and forced scenarios.

Screening accuracy per 1000 women

	$\bar{h} = 9.4 \mp 2.3\%$ (Data derived)			$\bar{h} = 15\%$ (Forced)		
**Age (modality)**	Correct	False positives	False Negatives	Correct	False positives	False Negatives
**25 (LBC)**	898 ∓ 3	94 ∓ 1	8 ∓ 3	900 ∓ 2	95 ∓ 1	5 ∓ 1
**25 (HPV-reflex)**	975 ∓ 4	15 ∓ 3	11 ∓ 3	978 ∓ 3	16 ∓ 3	7 ∓ 1
**35 (LBC)**	900 ∓ 2	95 ∓ 1	6 ∓ 2	901 ∓ 1	96 ∓ 1	4 ∓ 1
**35 (HPV-reflex)**	981 ∓ 3	12 ∓ 2	8 ∓ 2	983 ∓ 2	12 ∓ 2	5 ∓ 1
**45 (LBC)**	901 ∓ 2	95 ∓ 1	4 ∓ 2	901 ∓ 1	96 ∓ 1	3 ∓ 1
**45 (HPV-reflex)**	985 ∓ 2	9 ∓ 1	5 ∓ 2	989 ∓ 2	10 ∓ 2	3 ∓ 1
**55 (LBC)**	901 ∓ 2	96 ∓ 1	3 ∓ 1	902 ∓ 1	96 ∓ 1	2 ∓ 1
**55 (HPV-reflex)**	988 ∓ 3	8 ∓ 2	4 ∓ 1	989 ∓ 2	8 ∓ 2	2 ∓ 1

For peer review only

TRIPOD Checklist: Prediction Model Development

Section/Topic	Item	Checklist Item	Page
**Title and abstract**			
Title	1	Identify the study as developing and/or validating a multivariable prediction model, the target population, and the outcome to be predicted.	2
Abstract	2	Provide a summary of objectives, study design, setting, participants, sample size, predictors, outcome, statistical analysis, results, and conclusions.	2-5
**Introduction**			
Background and objectives	3a	Explain the medical context (including whether diagnostic or prognostic) and rationale for developing or validating the multivariable prediction model, including references to existing models.	2-3
	3b	Specify the objectives, including whether the study describes the development or validation of the model or both.	2-3
**Methods**			
Source of data	4a	Describe the study design or source of data (e.g., randomized trial, cohort, or registry data), separately for the development and validation data sets, if applicable.	3-4
	4b	Specify the key study dates, including start of accrual; end of accrual; and, if applicable, end of follow-up.	N/A
Participants	5a	Specify key elements of the study setting (e.g., primary care, secondary care, general population) including number and location of centres.	3-4
	5b	Describe eligibility criteria for participants.	3-4
	5c	Give details of treatments received, if relevant.	N/A
Outcome	6a	Clearly define the outcome that is predicted by the prediction model, including how and when assessed.	4-5
	6b	Report any actions to blind assessment of the outcome to be predicted.	N/A
Predictors	7a	Clearly define all predictors used in developing or validating the multivariable prediction model, including how and when they were measured.	3-5
	7b	Report any actions to blind assessment of predictors for the outcome and other predictors.	NA
Sample size	8	Explain how the study size was arrived at.	NA
Missing data	9	Describe how missing data were handled (e.g., complete-case analysis, single imputation, multiple imputation) with details of any imputation method.	NA
Statistical analysis methods	10a	Describe how predictors were handled in the analyses.	3-5
	10b	Specify type of model, all model-building procedures (including any predictor selection), and method for internal validation.	2-3
	10d	Specify all measures used to assess model performance and, if relevant, to compare multiple models.	3-7
Risk groups	11	Provide details on how risk groups were created, if done.	2-3
**Results**			
Participants	13a	Describe the flow of participants through the study, including the number of participants with and without the outcome and, if applicable, a summary of the follow-up time. A diagram may be helpful.	NA
	13b	Describe the characteristics of the participants (basic demographics, clinical features, available predictors), including the number of participants with missing data for predictors and outcome.	2-3
Model development	14a	Specify the number of participants and outcome events in each analysis.	NA
	14b	If done, report the unadjusted association between each candidate predictor and outcome.	NA
Model specification	15a	Present the full prediction model to allow predictions for individuals (i.e., all regression coefficients, and model intercept or baseline survival at a given time point).	5
	15b	Explain how to the use the prediction model.	3-5
Model performance	16	Report performance measures (with CIs) for the prediction model.	5
**Discussion**			
Limitations	18	Discuss any limitations of the study (such as nonrepresentative sample, few events per predictor, missing data).	6-7
Interpretation	19b	Give an overall interpretation of the results, considering objectives, limitations, and results from similar studies, and other relevant evidence.	6-7
Implications	20	Discuss the potential clinical use of the model and implications for future research.	6-7
**Other information**			
Supplementary information	21	Provide information about the availability of supplementary resources, such as study protocol, Web calculator, and data sets.	6-7
Funding	22	Give the source of funding and the role of the funders for the present study.	7

We recommend using the TRIPOD Checklist in conjunction with the TRIPOD Explanation and Elaboration document.
